# From Infection to Immunity: Understanding the Response to SARS-CoV2 Through *In-Silico* Modeling

**DOI:** 10.3389/fimmu.2021.646972

**Published:** 2021-09-07

**Authors:** Filippo Castiglione, Debashrito Deb, Anurag P. Srivastava, Pietro Liò, Arcangelo Liso

**Affiliations:** ^1^Institute for Applied Computing (IAC), National Research Council of Italy (CNR), Rome, Italy; ^2^Department of Biochemistry, School of Applied Sciences, REVA University, Bangalore, India; ^3^Department of Life Sciences, Garden City University, Bangalore, India; ^4^Department of Computer Science and Technology, University of Cambridge, Cambridge, United Kingdom; ^5^Department of Medical and Surgical Sciences, University of Foggia, Foggia, Italy

**Keywords:** COVID-19, *in-silico* modeling, virtual cohort, SARS-CoV-2, immunosenescence

## Abstract

**Background:**

Immune system conditions of the patient is a key factor in COVID-19 infection survival. A growing number of studies have focused on immunological determinants to develop better biomarkers for therapies.

**Aim:**

Studies of the insurgence of immunity is at the core of both SARS-CoV-2 vaccine development and therapies. This paper attempts to describe the insurgence (and the span) of immunity in COVID-19 at the population level by developing an in-silico model. We simulate the immune response to SARS-CoV-2 and analyze the impact of infecting viral load, affinity to the ACE2 receptor, and age in an artificially infected population on the course of the disease.

**Methods:**

We use a stochastic agent-based immune simulation platform to construct a virtual cohort of infected individuals with age-dependent varying degrees of immune competence. We use a parameter set to reproduce known inter-patient variability and general epidemiological statistics.

**Results:**

By assuming the viremia at day 30 of the infection to be the proxy for lethality, we reproduce in-silico several clinical observations and identify critical factors in the statistical evolution of the infection. In particular, we evidence the importance of the humoral response over the cytotoxic response and find that the antibody titers measured after day 25 from the infection are a prognostic factor for determining the clinical outcome of the infection. Our modeling framework uses COVID-19 infection to demonstrate the actionable effectiveness of modeling the immune response at individual and population levels. The model developed can explain and interpret observed patterns of infection and makes verifiable temporal predictions. Within the limitations imposed by the simulated environment, this work proposes quantitatively that the great variability observed in the patient outcomes in real life can be the mere result of subtle variability in the infecting viral load and immune competence in the population. In this work, we exemplify how computational modeling of immune response provides an important view to discuss hypothesis and design new experiments, in particular paving the way to further investigations about the duration of vaccine-elicited immunity especially in the view of the blundering effect of immunosenescence.

## Introduction

The global pandemic set up by the severe acute respiratory syndrome coronavirus 2 (SARS-CoV-2) in the early months of the year 2020 has reached considerable proportions and, to date, does not show signs of a slowdown when considered globally. In fact, as of 3:10pm CEST, 24 April 2021, there have been 145,216,414 confirmed cases of COVID-19, including 3,079,390 deaths, reported to WHO (1).

The mortality rates of the SARS-CoV-2 greatly differ across the globe, ranging from 0.8 to 9.2% (2), as a result of many factors including the ability to react to the pandemic by the various national health systems.

The COVID-19 disease has a quite variable clinical presentation: while the majority of individuals present with very mild disease, often asymptomatic, a few patients develop a life-threatening disease requiring intensive care. Recent review papers describing the characteristics of the virus SARS-CoV-2 and the disease COVID-19 can be found in (3). The strongest determinant of disease severity is age, with children presenting almost exclusively with mild disease, while individuals over 70 years of age are much more likely to develop severe COVID-19. This variation is likely due to both host and pathogen factors. Host factors may include differences in the immune response due to genetic determinants and immunological history. On the other hand, pathogen factors include transmission, entry and spread within the host, cell tropism, virus virulence, and consequent disease mechanisms.

To better understand what impact these factors may have in the differences observed in the host response to SARS-CoV-2, we set up the analysis of the dynamics generated by a computer model that considers both, the magnitude of the viral harm, and the subsequent innate and adaptive response set up to attempt achieving control of the infection. Thus, we used computer simulations to create a virtual cohort of infected individuals to study the effects on the pathogenesis of both host and pathogen factors. Note that this approach goes beyond the machine learning paradigm as the knowledge is generated through a set of equations/algorithms confirmed by the scientific literature and by past models. The simulation allows systems-level, multi-evidence analyses to simultaneously capture the dynamics of the major immune cell populations and the many protein mediators by which cells communicate, to sort out the determinants of disease severity.

## Simulating SARS-CoV-2 Course of Events in the Host

Up to date, there have been a large number of attempts to use mathematical and computational models to elucidate various aspects of the pandemic, from virus infection of the host to the pathogenesis of COVID-19 and the epidemiological aspects of viral spread in the population including the efficacy of containment measures (4). All kinds of modeling methodologies have been employed so far from classical differential equation models to describe the system dynamics to machine learning techniques to analyze available data (5).

The simulation model that we used in this study is a hybrid agent-based model for the simulation of the immune response to generic pathogens. It is equipped with elements of innate immunity consisting in macrophages, dendritic cells, natural killer cells, proinflammatory cytokines (e.g., IL-6, IL-12, IL-18, TNFa, IFNg), and of the adaptive immunity represented by B lymphocytes, plasma B antibody-producing lymphocytes, CD4 T helper and CD8 cytotoxic T lymphocytes. It is a polyclonal model as it embodies the primary sequences of binding sites of B-cell receptors (BCRs) and T-cell receptors (TCR), as well as the peptides and epitopes of the infectious agent (i.e., the SARS-CoV-2 in this case) (6).

The model (called C-ImmSim) represents a portion of, i) primary lymphatic organs where lymphocytes are formed and mature (i.e., mainly the red bone marrow and the thymus gland), ii) secondary lymphoid organs (e.g., a lymph node), which filters lymph, and where naïve B and T-cells are presented to antigens, and, iii) peripheral tissue which is dependent on the pathogen considered (in this case the lung).

While primary organs are just the source of lymphocytes equipped with a randomly generated receptor (actually only its complementarity-determining region, CDR), the secondary organs and the tissue are mapped onto a three-dimensional Cartesian lattice (however it is worth to specify that, since the initial condition, i.e., the initial cell counts, is uniform on the lattice and the diffusion is isotropic, spatiality does not really play a role in the present study). The thymus is implicitly represented by the positive and negative selection of immature thymocytes (7) before they enter the lymphatic system (8), while the bone marrow generates already immunocompetent B lymphocytes.

C-ImmSim incorporates several working assumptions or immunological theories, most of which are regarded as established mechanisms, including the clonal selection theory of Burnet (9, 10), the clonal deletion theory (e.g., thymus education of T lymphocytes) (7, 11), the hypermutation of antibodies (12–14), the replicative senescence of T-cells, or the Hayflick limit (i.e., a limit in the number of cell divisions) (15, 16), T-cell anergy (17, 18) and Ag-dose induced tolerance in B-cells (19, 20), the danger theory (21–23), the (generally unused) idiotypic network theory (24, 25).

Being a general-purpose modeling platform, C-ImmSim lends itself to characterize the role of the immune response in different human pathologies. For instance, in simulating viral infections such as HIV we have depicted the evolutionary path of the wild type virus inside the host due to its high replication rate (26); in the case of EBV infection, we have shown that the ability of the virus to establish long term persistence is dependent on access of latently infected cells to the peripheral pool where they are not subject to immunosurveillance (27). While simulating cancer immuno-prevention we have shown, as supported by *in vivo* mice experiments, that the humoral response is fundamental in controlling the tumor growth (28, 29). In a study of type 1 hypersensitivity we have elucidated the role of timing and dosage in the administration of anticancer drugs with respect to the risk of having an allergic reaction (30). While reproducing the pathogenesis of type 2 diabetes we have pinpointed the deleterious effects of a chronic inflammation (31). The model has also been used to describe specific aspects of the immune dynamics such as lymphocytes homing in lymph nodes (32), the gene regulation leading to cell differentiation (33), the clonal dominance in heterologous immune responses (34) and also vaccination-eliciting fish immunity (35). Finally, and relevant to the present study, the model has recently been used to test in silico the response to a multi-epitope vaccine against SARS-CoV-2 (36, 37).

In C-ImmSim each simulated time step corresponds to eight hours of real life. Cells diffuse randomly in the represented volume and interact among them. Upon specific recognition through receptor bindings, they perform actions that determine their functional behavior. These probabilistic rules define the transition of the entities from one “condition” to another. Each rule is executed only if the involved parties are in enabling states (e.g., naïve, active, resting, antigen-presenting).

Besides cell-cell interaction and cooperation, the model simulates the intra-cellular processes of antigen uptake and presentation. Endogenous antigens are fragmented and combined with MHC class I molecules for presentation on the cell surface to CTLs’ receptors (this is the cytosolic pathway), whereas exogenous antigens are degraded into small pieces, which are then bound to MHC class II molecules for presentation to T helpers’ receptors (this is the endocytic pathway).

The stochastic execution of the algorithms that code for the dynamical rules of the automaton, results in a sequence of cause/effect events culminating in the production of effector immune cells and setup of immunological memory. The starting point of this series of events is the injection of an antigen which, here, consists of the virus. This may take place any time after the simulation starts (the sequence of events of the SARS-CoV-2 simulation is reported in **Appendix A of the Supplementary Materials**). Initially, the system is “naïve” in the sense that there are neither T and B memory cells nor plasma cells and antibodies. Moreover, the system is designed to maintain a steady-state of the global population of cells (homeostasis of the normal peripheral blood leukocyte counts), if no infection takes place.

Besides the parameters defining the characteristics of the virus related to attachment, penetration, replication, and assembly (i.e., its fitness), the SARS-CoV-2 virus in this model is defined as a set of B-cell epitopes and T-cell peptides consisting of amino acid 9-mers and defining its antigenicity. If the infection is stopped or becomes persistent or even kills the virtual patient it depends on the dose of the virus, its fitness, and the strength of the immune response aroused. All of these variables determine if, and to what degree, the success of the immune system requires the cooperation of both the cellular and the humoral branch, as shown in past simulation studies (38).

**Figure 1:**
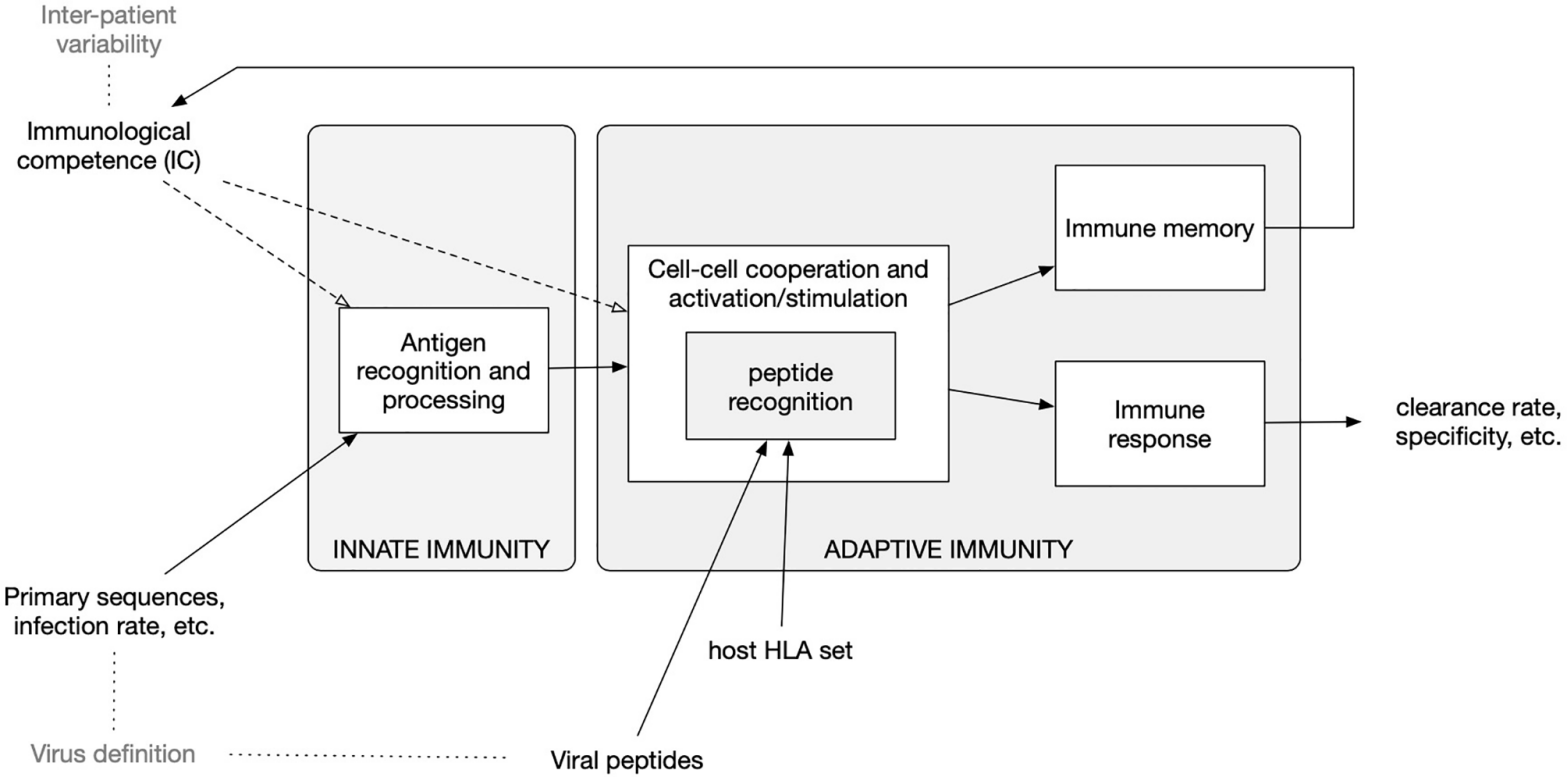
Diagram of the *in-silico* model components and accepted input. The model embodies functions to calculate the clonal affinity to precomputed viral peptides of the selected pathogen (defined by its primary sequence) with respect to a specific Human Leukocyte Antigen (HLA) set. The population-dynamics of the elicited lymphocytes clones, resulting from the infection by the SARS-CoV-2, provides a varying degree of efficiency of the immune response which, as it turns out, correlates with the parameters defining both the immunological competence (IC) of the virtual host and the virus definition.

To improve the peptide-prediction performance, the most important difference to the previous version of the model (39) is that, rather than using position-specific-score-matrices (PSSM) to weight the binding contribution of the amino-acids composing the protein segments in the bounds (40, 41), we resort to pre-computed ranked lists of T-cell epitopes calculated with the original neural network NetMHCpan method (42–44). This feature, which is described below, follows the choice of a specific Human Leukocyte Antigen (HLA) set as described in the next section. A diagram of the model components is shown in **Figure 1**.

### Selecting the HLA Haplotype

The C-ImmSim model accounts for differences in the HLA haplotype when determining which peptides are presented by antigen-presenting cells. To this end, for each HLA molecule, it takes in input a list of such peptides together with a propensity of each of them to bind to the HLA. This list is computed by using third-party immunoinformatics tools as described in the next section *(Computing the peptide immunogenicity)*.

The “HLA haplotype freq search” in the “Allele Frequency Net Database” (45) was used to select two HLA-A, two HLA-B and two DRB alleles which are most prevalent in the US population (46). The result pointed to the following alleles: HLA-A*02:01, HLA-A*24:02, HLA-B*35:01, HLA-B*40:02, DRB1*07:01, and DRB1*15:01.

### Computing the Peptide Immunogenicity

The strain of SARS-CoV-2 used in this study corresponds to the reference sequence *NCBI Reference Sequence: NC_045512.2*. The primary structure of these proteins has been used to identify cytotoxic T peptides (CTL peptides) and helper T peptides (HTL peptides). To this aim, we have employed two immunoinformatics tools. In particular, for the definition of CTL epitopes, the “ANN 4.0 prediction method” in the online tool MHC-I binding prediction of the *IEDB Analysis Resource* (47) was used for the prediction of 9-mer long CTL peptides which had an affinity for the chosen set of HLA class I alleles (i.e., HLA-A*02:01, HLA-A*24:02, HLA-B*35:01 and HLA-B*40:02) (48–50). The peptides were classified as strong, moderate, and weak binders based on the peptide percentile rank and IC50 value. Peptides with IC50 values <50 nM were considered to have high affinity, <500 nM intermediate affinity, and <5000 nM low affinity towards a particular HLA allele. Also, the lower the percentile rank, the greater is meant the affinity (48–50). The list of peptides is reported in **Appendix B of the Supplementary Materials**.

For what concerns the HTL peptides, the NetMHCIIpan 3.2 server (51) was used for the prediction of 9-mer long HTL peptides which had an affinity for the HLA class II alleles (i.e., DRB1*07:01 and DRB1*15:01) used in this study (52). The predicted peptides were classified as strong, intermediate, and non-binders based on the concept of percentile rank as given by NetMHCII pan 3.2 server with a threshold value set at 2, 10, and >10%, respectively. In other words, peptides with percentile rank ≤2 were considered as strong binders whereas a percentile rank between 2 and 10% designate moderate binders; peptides with percentile score >10 are considered to be non-binders (52). The list of CTL and HTL peptides and the relative affinity score is reported in **Appendix C of the Supplementary Materials**.

### Quantifying the Immunological Competence

It is widely accepted that aging is accompanied by remodeling of the immune system. With time, there is a decline in overall immune efficacy, which manifests itself as an increased vulnerability to infectious diseases, a diminished response to antigens (including vaccines), and a susceptibility to inflammatory diseases. The most important age-associated immune alteration is the reduction in the number of peripheral blood naïve cells, accompanied by a relative increase in the frequency of antigen-specific memory cells. These two alterations are extensively reported in the literature and account for the immune repertoire reduction (53, 54). Along with the process called “inflammaging”, the reduction of immune repertoire is considered the hallmark of immunosenescence (55).

**Figure 2:**
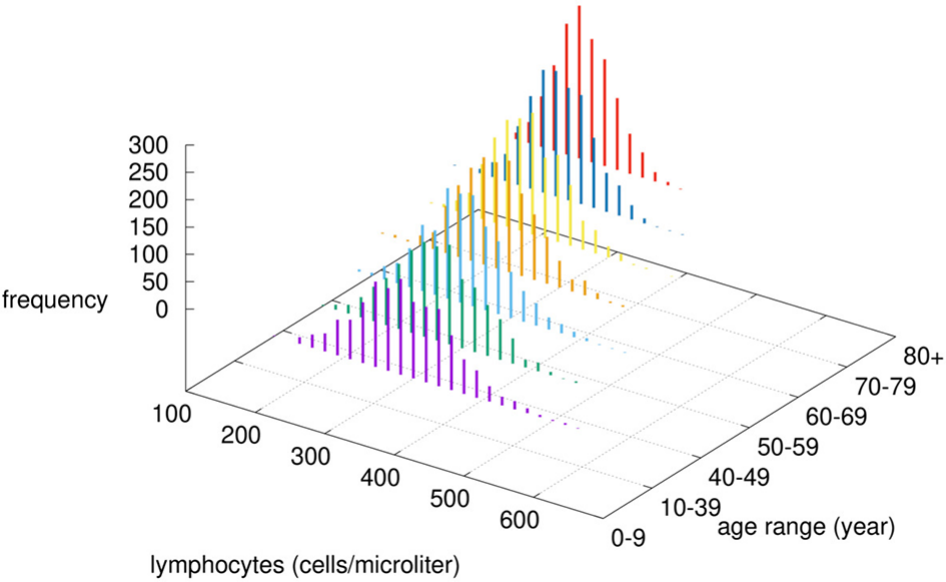
Modified lymphocytes counts by age-class. Given the chosen value of *α*, the immunological competence is less than one, therefore by increasing the age, the cell numbers are drawn by a normal distribution with reduced mean value *μ* · *IC* (*age*) and reduced variance [*σ* · *IC*(*age*)]^2^.

To model the reduction of immune efficacy we first defined the parameter “immunological competence” *IC*∈(0,1] and assumed it in a simple linear relationship with age. Specifically, we set *IC* ≡ *IC*(*age*) = –*α age* + 1 with the value of the parameter *α* = 45 · 10^-4^ determined using epidemiological data as described below. Given the age, the parameter *IC* is then used to modulate both innate and adaptive immunity as follows: i) the phagocytic activity of macrophages and dendritic cells, represented by a probability to capture a viral particle, is rescaled respectively as *p_M_* = *IC* · *u* and *p_DC_* = *IC* · *v*, where *u*~*U*_[*a*,*b*]_ and *v*~*U*_[5×_ *_a_*_,5×_ *_b_*_]_ are two random variables uniformly distributed in the ranges [*a*, *b*] and [5 · *a*, 5 · *b*] with *a* = 25 × 10^-4^ and *b* = 10^-2^; ii) as for the adaptive immunity it is adjusted according to the immunological competence parameter *IC* by decreasing the lymphocyte counts (hence B, Th, and Tc) to reflect a reduction in the repertoire of “naïve” cells with immunological history due to accumulation of memory cells filling the immunological compartment. In particular, the number of white blood cells *N* is computed as $N∼IC\cdot N\left(\mu ,\sigma^{2}\right)=N\left(IC\cdot \mu ,IC^{2}\cdot \sigma^{2}\right)$ where $N\left(\mu ,\sigma^{2}\right)$ is a normal distribution with average *μ* and standard deviation *σ* (for each lymphocyte type B, Th and Tc) chosen to reflect the reference leukocyte formula for an average healthy human adult (see **Figure 2**) (56).

### Adapting the Model to SARS-CoV-2 Characteristics

The infection and the dynamical features of the SARS-CoV-2 viral strain have been characterized by two parameters: i) *V*_0_ corresponding to the infectious viral load at time zero, and, ii) the affinity of the virus spike protein to the ACE2 receptor on target cells, called *p_A_*. In particular, $V_{0}∼U_{\left[c_{1},d_{1}\right]}$has been taken randomly in the interval [*c*_1_ = 5, *d*_1_ = 5 · 10^5^], while $p_{A}∼U_{\left[c_{2},d_{2}\right]}$ in the interval [*c*_2_ = 10^-3^, *d*_2_ = 10^-1^].

Upon choice of the age-class determining the immunocompetence value *IC*(*age*) hence *p_A_* and *p_DC_* as well as the lymphocyte counts, the simulations depict the immune-virus competition eventually culminating in a successful, or not, virus-clearing response controlling its growth. Sometimes this control is not perfectly efficient. In those cases, the result is a longer viral persistence possibly going much beyond the length of the observation period of 30 days (cf. **Figure 4**).

The sequence of events from viral infection leading to a full-fledged immune response is detailed in **Appendix A of the Supplementary Materials**. At each time step of the simulation, C-ImmSim dumps all variables allowing for a detailed analysis of the dynamics. A full output example of a simulation is reported and described in **Appendix D of the Supplementary Materials**.

## Modeling a Representative Cohort of Infected Individuals

We have simulated a large number of infections (1500 for each age-class for a total of 10500 independent simulations) by varying the parameters identifying both the viral characteristics and the individual immunological competencies. The seven age classes considered were 0-9, 10-39, 40-49, 50-59, 60-69, 70-79 and 80+. As specified above, the age-class determines the immunocompetence parameter *IC* which, in turn, sets *p_M_* and *p_DC_* as well as the lymphocyte counts in the *in-silico* individual, we can characterize each simulation by the set of parameters (*IC*(*age*), *V*_0_, *p_A_*). Moreover, due to the stochasticity of the model depending on the random number realizations, each simulation corresponds to a different trajectory in the space of the variables. It follows that each simulation coincides with an *in-silico* patient with variable immunological characteristics (*IC*) and, at the same time, infected by a slightly different viral burden (*V*_0_ and *p_A_*).

The intervals within which these parameters vary have been chosen to reproduce the age-class incidence of disease severity of infected individuals. The age-class incidence varies wildly among regions mostly due to a different definition of COVID-19 related deaths. Moreover, due to the lack of confirmation of the causes of death in many cases in periods of high emergency as that of March/April 2020 in Italy, these rates should not be considered strictly but rather indicative of the negative exponential-like relationship of the death incidence with age. Reference values we used were the fatality rates from the Chinese Center for Disease Control and Prevention (CDC) as of 17th February, the Spanish Ministry of Health as of 24th March, the Korea Centers for Disease Control and Prevention (KCDC) as of 24th March, and the Italian National Institute of Health, as presented in the paper by Onder et al. (57) as of 17th March (57, 58).

To reproduce this age-related incidence of *in-silico* cases we linked the simulated viral load at a certain time to the clinical status (*clinical endpoint*). This has been done according to the rationale that a patient whose viral load is still quite high after thirty days from infection can be considered at a very high risk of death. In fact, in most mild cases, the clinical signs and symptoms (mostly fever and cough) have been reported to resolve within 3 weeks from the diagnosis (which translates into approximately 30 days from infection). Instead after 3 weeks, several authors have described severe cases with progressive multi-organ dysfunction with severe acute respiratory dyspnea syndrome, refractory shock, anuric acute kidney injury, coagulopathy, thrombocytopenia, and death (59).

### Stratifying the *In-Silico* Cohort of Patients

The analogy of some simulation variable to a realistic *clinical endpoint* allows stratifying the in-silico patients for a more concrete interpretation of the results. Based on the viral load observed at day 30 (indicated by *V*_30_) and a threshold *θ* we assume to identify the virtual patients in one of the three following classes:

- Critical: if *V*_30_ > *θ*, namely, the viral load at day 30 is still high; this class includes weak and late responders;
- Partially recovered: those who are still positive but have a low viral load, meaning that the immune response is controlling the viral replication (i.e., 0 < *V*_30_ ≤ *θ*); note that this class includes the asymptomatics;
- Fully recovered (or just Recovered): those who have cleared the virus (i.e., *V*_30_ = 0).

**Figure 3:**
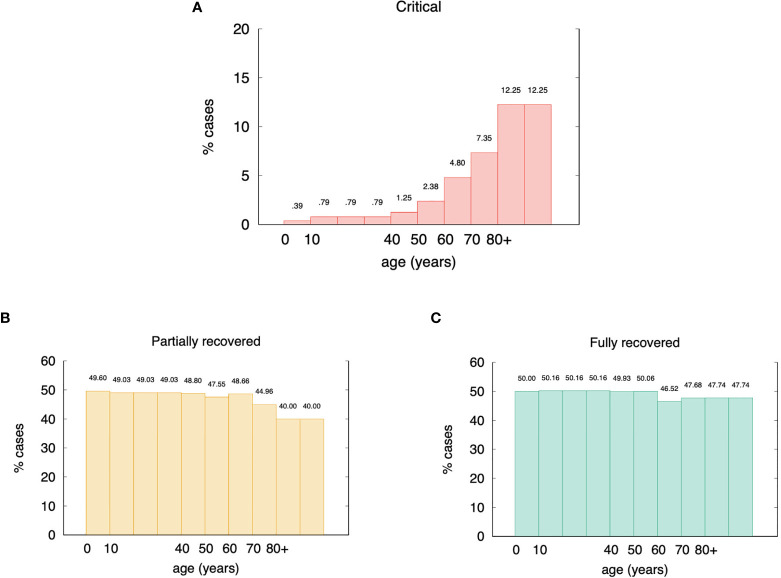
Age incidence of stratified *in-silico* patients (*θ* = 120 virions per micro liter). Panel **(A)** shows the percentages of critical cases broken down in age classes. Overall they account for 4.3% of the total virtual individuals. Panel **(B)** shows the partially recovered ones for a gross 46.8%, and panel **(C)** shows the remaining 48.8% classified in the recovered cases class. These figures are in good agreement with epidemiological statistics (57).

According to this assumption/definition, by choosing the cutoff *θ* =120 viral particles per micro-liter of simulated volume, we obtain the stratification of the virtual individuals shown in **Figure 3A**. Of all *in-silico* individuals, we get 4.3% of critical cases (broken down in age classes in **Figure 3A**), 46.8% of partially recovered (**Figure 3B**), and 48.8% recovered cases (**Figure 3C**). These figures sound very much in line with current epidemiological statistics when considering that the recovered cases here simulated include asymptomatics (57).

This result shows an interesting and surprisingly high fraction of in-silico patients in the partially recovered class. This class includes patients that, at the end of the simulated period of thirty days, are still positive albeit manifesting an active immune response, regardless of being asymptomatic or not. This question is discussed below.

**Figure 4:**
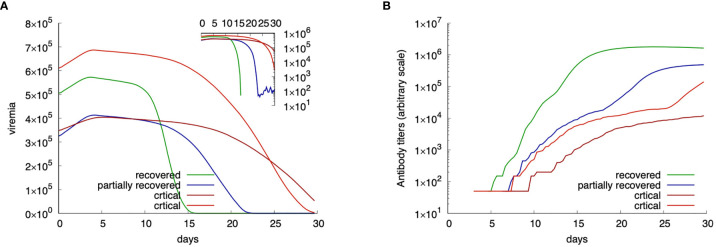
Examples of four *in-silico* cases with different outcome. Panel **(A)** shows the viral load while panel **(B)** the corresponding antibody titers. Red lines show critical cases; blue a partially recovered case; green a fully recovered case.

These special cases can be better examined in **Figure 4**, which shows four distinct exemplifying runs with different outcomes. In **Figure 4A** the viremia is shown as a function of time. Red lines correspond to individuals who reach the critical condition *V*_30_ > *θ* thus falling in the class “critical”. The green line corresponds to a viral clearance corresponding to a fully recovered case, and the blue line shows a situation in which the virus is not completely cleared but stays below the threshold value *θ*. This case corresponds to one of what we call partially recovered as it represents virtual individuals that produce an immune response (cf. same figure, **Figure 4A** showing the corresponding antibody titers) which turns out to be insufficient to clear the virus. These “unresolved infections” include asymptomatic cases and are worth the further analysis described below.

To note that the two examples of critical outcomes (red curves) originate from a quite different initial viral load. Also, to note that the fully recovered (green) case starts with a viral load that is higher than one critical case, still the immune response manages to control the infection. The blue curve shows a partially recovered case which greatly decreases the viral load (inset plot of **Figure 4A**) but does not clear it completely.

### How the Model Explains Symptoms

It is worth clarifying that the term “symptom” has no meaning in the *in-silico* framework unless a link between model variables and possible *clinical endpoints* is drawn. Also, we should note that we have no concept of comorbidity here that would help in defining the “status” of the virtual patient. To overcome this limitation, besides the viral load at day 30, we think up the following quantities (or variables) as *clinical endpoints*: (a) the damage in the epithelial compartment, namely, percent of virus-target cells that are dead at the time of observation as a surrogate marker of vascular permeability; (b) the concentration of pyrogenic cytokines as a *surrogate marker* of fever, i.e., Prostaglandins TNFa, IL-1, and IL-6 causes fever people get varying degree of severity with COVID-19.

**Figure 5:**
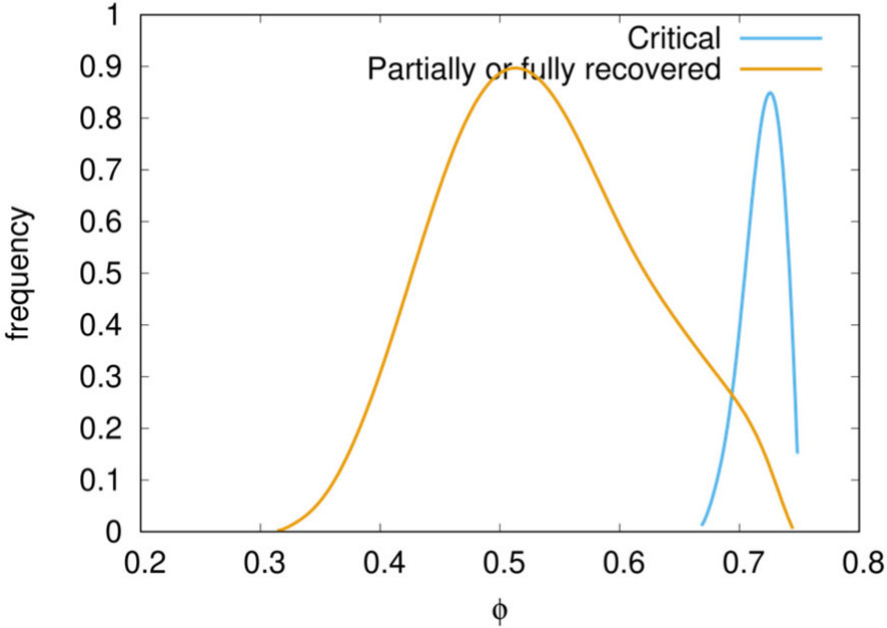
Distribution of epithelial damage *φ* of the cohort cases classified in critical and non-critical. A delimiting value *φ_c_* = 0.63 separates well the two classes.

Of these two potential surrogate markers of criticality, the first appears more appropriate. While the amount of pyrogenic cytokines (surrogate clinical endpoint b.) correlates with the severity of the disease, the most striking difference in the critical cases versus non-critical (i.e., recovered plus partially recovered) is seen when comparing the accumulated damage in the epithelial compartments (*φ*) computed as the fraction of depleted epithelial cells due to the immune cytotoxicity of SARS-CoV-2 infected cells during the whole observation period (surrogate clinical endpoint a.),

(1) $$\phi =1-\frac{1}{T\cdot E(0)}\int_{T}^{0}E(t)dt$$

where *E*(*t*) is the epithelial count per microliter of simulated volume and *T* is the time horizon of 30 days (note that *φ* ∈ [0,1]). Indeed, when we plot the distribution of *φ* for the critical and non-critical cases (i.e., partially recovered plus fully recovered) we obtain what is shown in **Figure 5**. The plot clearly shows that for critical in-silico patients the damage is much more pronounced than for non-critical ones. This prompt us to use the threshold *φ_c_* = 0.63 to set apart patients who have mild infections [about 80% as in reality (60–62)] to those having a severe disease [15% with dyspnea, hypoxia, lung changes on images (63)] or critical illness [5%, respiratory failure, shock, multi organic dysfunction, cytokine storm syndrome (64)]. Therefore, we label patients with *φ* ≥ *φ_c_* as symptomatic while those with *φ < φ_c_* asymptomatic. According to this further stratification patients who are still positive (i.e., 0 ≤ *V*_30_ < *θ*) and have no symptoms (i.e., *φ < φ_c_*) account for about 44% of the simulations which is in line with current estimates of asymptomatic incidence (65).

### The Model Indicates That a High Infective Viral Load Carries a Serious Risk

We tested the correlation between the antigen abundance (or infective viral load *V*_0_) and the severity of the infection. The Mann-Whitney-Wilcoxon (MWW) test shows a significant (i.e., p-value< 10^-3^) difference between infecting viral load *V*_0_ in the three classes critical, partially recovered, recovered. In particular, we find that a higher *V*_0_ is a strong correlate of disease severity (66). Of interest is the fact that there is no significant difference among age groups, that is, *V*_0_ is not predictive of the disease severity for the age (67) (MWW test p-value>0.05).

### Simulated IL-6 Correlates With Disease Severity but Youngers Generate More

Significantly, in most critically ill patients, SARS-CoV-2 infection is associated with a severe clinical inflammatory picture based on a severe cytokine storm that is mainly characterized by elevated plasma concentrations of interleukin 6 (68). In this scenario, it seems that IL-6 owns an important driving role in the cytokine storm, leading to lung damage and reduced survival (69).

**Figure 6:**
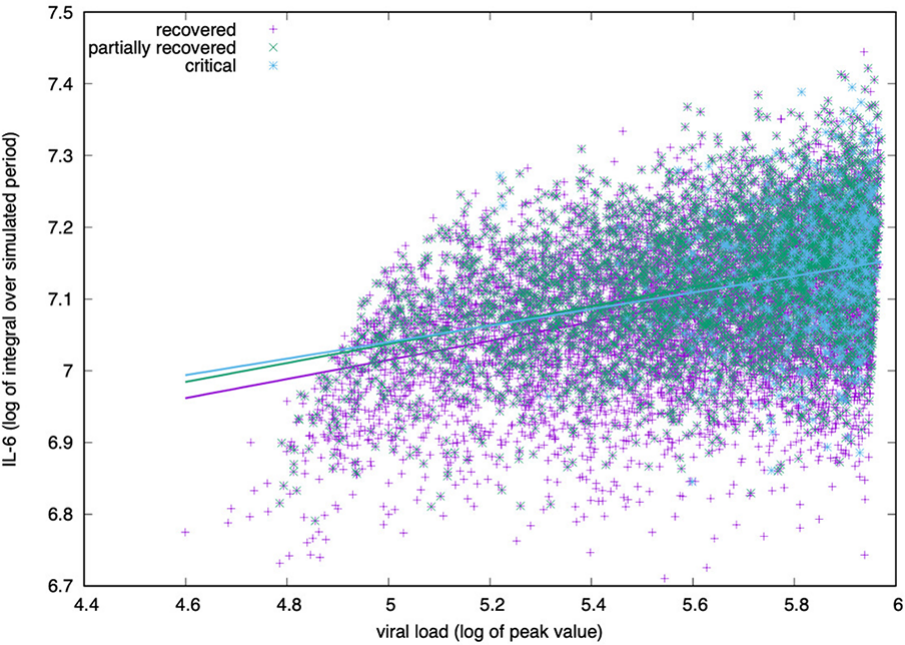
IL-6 concentration (log-scale) correlates positively with the viral load measured at the peak (i.e., its maximum value during the simulated period). The correlation is positive for all groups, critical, partially recovered and recovered with no significant difference in the degree of correlation.

The simulation agrees with this finding as reported in **Figure 6**. Plotting the peak value of the viral load (i.e., the maximum value attained in the observed period) versus the logarithm of the integral of IL-6 over the whole period (cf. eq(2) in next section 3.5), we see a positive correlation regardless of the outcome.

**Figure 7:**
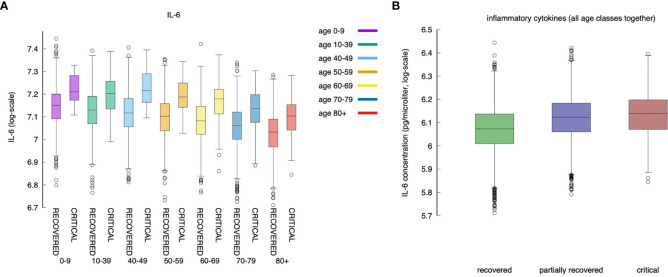
IL-6 (measured as the area under the curve, as defined in section *Younger In Silico Individuals Deal With the Virus by Producing More Cytokines*) to age and severity of the disease. Panel **(A)** shows the difference in IL-6 between critical and recovered as a function of age. Panel **(B)** shows the same information for all severity classes, lumping up the age. Inflammation correlates positively with severity of the disease (MWW test, p-value < 10^{-3}) (70).

For all age classes, as shown in **Figure 7A**, a critical clinical course is associated with a significantly higher concentration of pro-inflammatory cytokine IL-6. **Figure 7B** shows the same information for all age classes lumped together. The cytokine concentration on the y-axis is calculated as the integral over the whole simulated period (definition in eq(2) of section 3.5). The positive correlation between inflammation and severity of the clinical course is a consequence of the struggle of the immune system to cope with the infection. However what **Figure 6** reports is a generic higher production of IL-6 in younger individuals compared to elders. The explanation of this outcome becomes visible following the line of consequences starting from a stronger cytotoxic activity (see **Figure 12B** below) that killing infected cells cause a stronger release of danger signal to which macrophages respond by secreting IL-6. Since younger have a higher immunological competence (IC), they respond with both stronger cytotoxic response and better innate (i.e., macrophage) activity. The result is the somehow counterintuitive observation that while younger individuals have a higher degree of inflammation, they report a smaller propensity to experience severe outcomes.

### Younger *In Silico* Individuals Deal With the Virus by Producing More Cytokines

**Figure 8:**
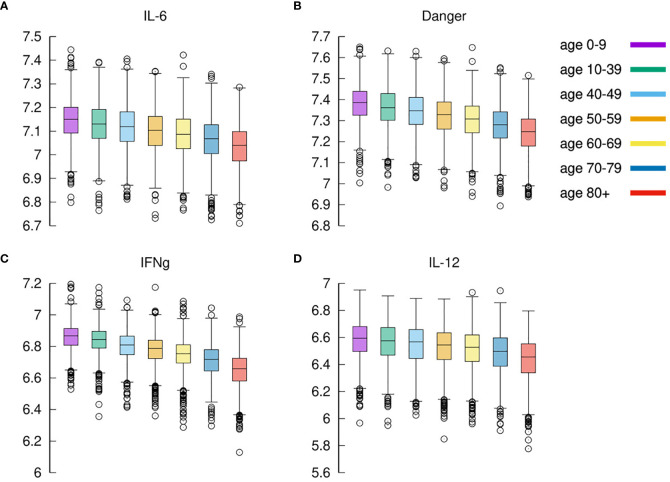
Cytokines. Panel **(A)** shows IL-6 (cfr. **Figure 7A** summing disease severity classes); panel **(B)** shows Danger signal; panel **(C)** shows IFNg, and panel **(D)** shows IL-12. Each plot shows the cumulative value of the cytokine in the whole observation period per each age-class. All panels exhibit the same reduction for increased age.

What is observed in the production of IL-6 in younger individuals extends to all cytokines produced during the response to the inflammation. We find that, in general, cytokines’ cumulative production during the whole simulated period correlates inversely with the age. Calling *c_x_* the concentration of cytokines *x* in the simulated volume, where *x* is one of IL-6, D, IFNg, and IL-12, we define

(2) $$\sigma_{x}=\int_{0}^{T}c_{x}\left(t\right)dt\;$$

the cumulative value of cytokines in the whole observation period. **Figure 8** shows *σ_IL_*_6_, *σ_D_*, *σ_IFNg_*, and *σ_IL_*_12_ against age. What **Figure 8** shows is that there is a clear reduction of cytokines’ production with age. This, similarly to what was discussed in the previous section, is due to the reduced immune activity which indeed is determined by a reduced immunological competence with the age (71). To justify the apparent contrast with the fact that elder acute infected individuals are more prone to experience a cytokine storm, we should openly regard one of the limitations of the model, namely, the lack of further cytokine feedbacks that are activated during extended pneumonia.

### Model Predicted IFNg Concentration Is Higher in Milder Courses of the Infection

**Figure 9:**
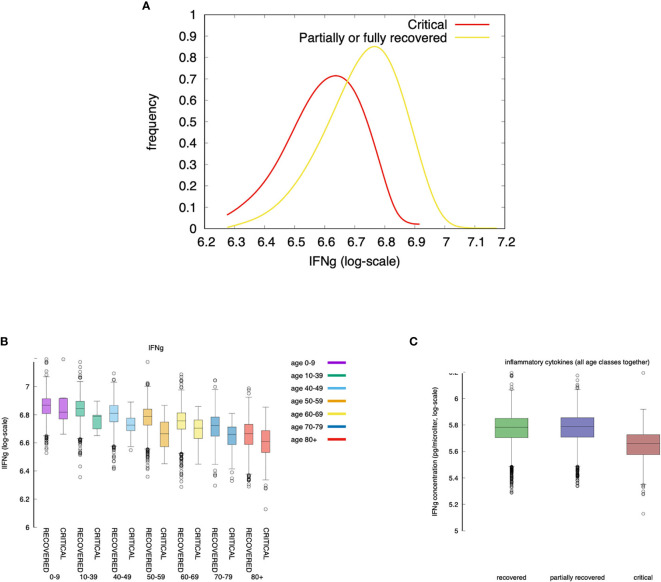
Severe cases are associated to a lower concentration of IFNg. IFNg is measured as the area under the curve (i.e., the integral in the simulation time window). Panel **(A)** shows that the concentration of IFNg is lower in severe cases than in moderate cases. This is in agreement with (72). When summing all age classes **(C)** IFNg shows an inverse correlation with disease severity. The same is observed when looking at each age group separately **(B)**. Interestingly, recovered and partially recovered do not show a meaningful difference when compared to the critical cases **(C)**.

The expression of IFNg by CD4 tends to be lower in severe cases than in moderate cases as shown in **Figure 9A**. This is in agreement with (72).

The inverse correlation of interferon-gamma (IFNg) with disease severity is observed in all age groups (**Figure 9A** and also in **Figure 9C** when summing all age classes). Interestingly, recovered and partially recovered do not show a meaningful difference when compared to the critical cases (**Figure 9C**).

IFNg is released by natural killer (NK) cells upon bystander stimulation by danger signals (Rule n.5 in **Appendix A of Supplementary Materials**) which, in turn, is released by infected/injured epithelial cells upon viral infection (Rule n.3) and when killed by cytotoxic cells (Rule n.18). This means that in young individuals a prompt activation of NK cells due to higher immunological competence, and a stronger cytotoxic response killing infected cells, are sufficient to control the “acute” production of danger signal impacting on the production of IFNg.

### *In Silico* Cytokine Storm Goes With Symptoms

**Figure 10:**
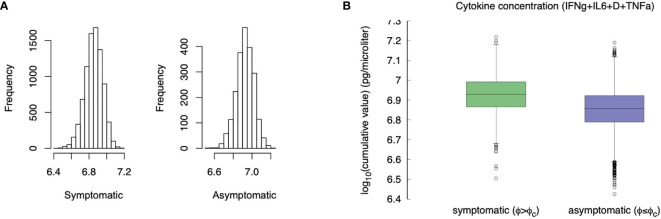
Compare the “cytokine storm” in the two groups asymptomatic *φ* < *φ_c_* and symptomatic *φ* ≥ *φ_c_*. Panel **(A)** shows the histogram, panel **(B)** compares the whiskers. The difference is statistically significant (p-value< 10^-3^).

If we use the cumulative value of inflammatory cytokines as a variable, namely, *σ_IFNγ_ + σ_IL_*_6_ + *D* + *σ_TNFa_* (i.e., the sum of the integrals) and we use the accumulated damage in the epithelial compartments, that is, the fraction of depleted epithelial cells due to the immune cytotoxicity *φ* defined in eq(1) (cf. section 3.2) as the discriminating criteria between symptomatic and asymptomatic, we observe what is shown in **Figure 10**.

Compared with asymptomatic cases, the symptomatic ones more frequently have markedly higher levels of inflammatory cytokines. The difference of the virtual patients in the two classes is statistically significant (MWW test, p-value< 10^-3^), which is in line with the clinical finding of higher inflammatory levels in severe disease progressions (72). This result seems to contrast what is stated in section 3.5, namely that younger individuals produce more cytokines but have a less severe course of the disease. However, the explanation provided by the simulation is that those who deal with the infection more rapidly (including asymptomatic) produce, overall, a smaller amount of cytokines and therefore have a lower risk of “cytokine storm”. On the other hand, an inconclusive immune response leads to chronic inflammation, hence pronounced symptoms (e.g., extended epithelial damage) and ultimately in a cytokine storm.

### Why Is the Predicted Immune Response Quicker in Younger Individuals?

It has been suggested that in younger individuals several factors contribute to the lower numbers of patients observed with severe disease, namely: lower number of ACE receptors, overlapping immunity against coronaviruses, and a more efficient intact immune system.

**Figure 11:**
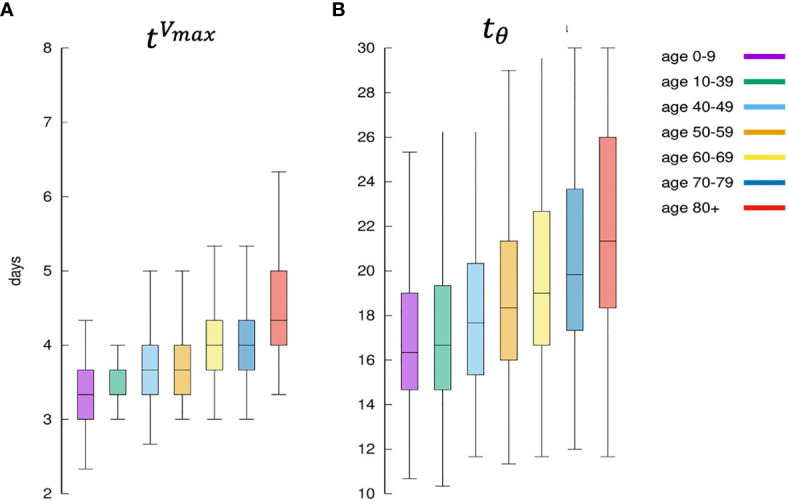
Panel **(A)** shows for each age class, the days elapsed from infection until the viral load starts to decrease. This is indicated as $t^{V_{max}}=\max_{t}\left\{V\left(t\right)\right\}$since it means the distribution of the time when the antigen reaches the peak. It is a measure of how the immune defenses are mobilized, hence its speed. Panel **(B)** shows the corresponding distribution of $t_{\theta }=\min_{t}\left\{V\left(t\right)\leq \theta \right\}$namely the time it takes for the immune response to bring the viral load to fall below *θ*. It is a measure of the efficiency of the immune response in clearing the infection.

**Figure 11** shows that the immune response is quicker in younger virtual individuals compared to elder ones. The speed of the immune response is calculated in terms of the time [in days) the viral load *V*(*t*) reaches its maximum [indicated $t^{V_{max}}$where *t*:*V*(*t*) = *V^max^* and $V^{\text{max}}=\underset{t}{\max}V(t)$] and starts to decline due to the immune response. **Figure 11A** shows the distribution of $t^{V_{max}}$for each age class. Younger individuals develop a faster response and consequently, the virus is cleared earlier. This is shown in **Figure 11B** which plots the distribution of the time (in days) it takes the immune response to decrease the viral load below the threshold *θ* whenever this happens (the cases for which *V*(*t) > θ,∀t*, are not counted in this statistics). **Figure 11B** is in line with the fact that younger individuals mount a quicker immune response that is generally more efficient than those in elder people thus eradicating the virus in a shorter time.

### The Key Role of the Humoral Response

**Figure 12:**
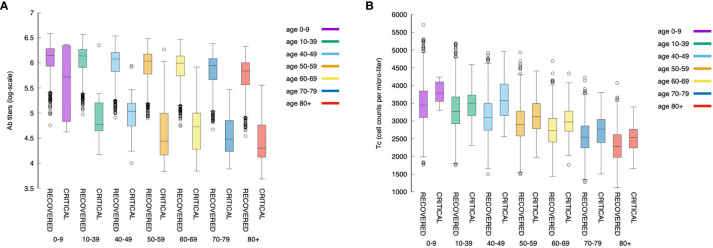
The relationship between the magnitude of the immune response and age is maintained also when looking at antibodies and cytotoxic cells. Panel **(A)** shows the antibody titers for all age classes comparing recovered with critical patients. Panel **(B)** shows cytotoxic cell (Tc) counts (peak values).

**Figure 12** shows that younger individuals have a higher production of antibodies when compared to elder individuals. This is evident for both critical and recovered (partial or fully recovered) individuals. However, the most striking observation when considering the difference between recovered and critical cases is the gap in antibody titers present in virtually all age classes (**Figure 12A**). This indicates a strong protective role of the humoral response making a split between recovered and critical patients.

**Figure 12B** shows the corresponding statistics for the cytotoxic T cell (peak value) count per age-class and critical status. This plot consistently evidences that youngers have a stronger response than elders. Interestingly, in contrast to the humoral response, in all age classes, the cytotoxic response in critical individuals is higher than in recovered ones revealing the attempt of the immune system to counterbalance the inefficacy of the humoral response.

**Figure 13:**
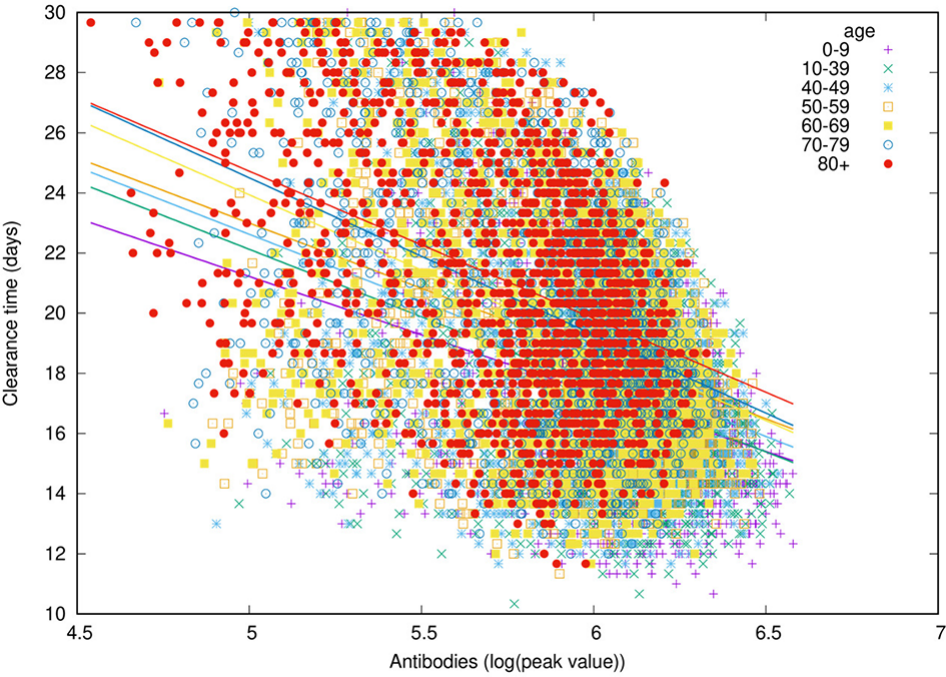
The peak value of antibody titers correlates inversely with the time-to-clear-virus *t_θ_*.

Moreover, a further view at the antibody titers reveals that its peak value [i.e., $V^{max}=\underset{t}{\max}V\left(t\right)$] correlates inversely with the clearance time (*t_θ_*), that is, faster responses are obtained with lower production of antibodies (cf. **Figure 13**). This is in line with the hypothesis that asymptomatic individuals develop a rapid but mild response which clears the infection (73).

It should be noted, however, that there are still substantial uncertainties on the data available due to the variable diagnostic accuracy of different serological tests for COVID-19, therefore more well-designed large clinical studies are warranted to address this matter (74, 75). Interestingly, the same cannot be said when considering peak values of Tc counts, that is, the cytotoxic response does not correlate, either positively or negatively, with time-to-clearance (not shown).

### Antibody Titers Have a Prognostic Value After Day 25

We have used a logistic regression classification to see if by measuring the antibody titers and the CTL counts at day *t* < 30 we can infer the outcome at the end of the simulated period of *t* = 30 days. We call *V*(*t* = 30) = *V*_30_ the viral load on day 30 after the infection.

Formally, the logistic regression classifier uses the data set {(*x_i_*, *y_i_*)}_i=1…_ *_m_* where $x_{i}=\left(x_{i}^{\left(1\right)},x_{i}^{\left(2\right)}\right)$is the feature vector consisting of the normalized cytotoxic T-cell lymphocytes count, $x_{i}^{\left(1\right)}=Tc\left(t\right)$and the antibody titers at day *t*, $x_{i}^{\left(2\right)}=Ab(t).$While *y_i_* = 0 if the corresponding run has *V*_30_ ≤ *θ* and *y_i_* = 1 if the corresponding runs has *V*_30_ > *θ*.

**Figure 14:**
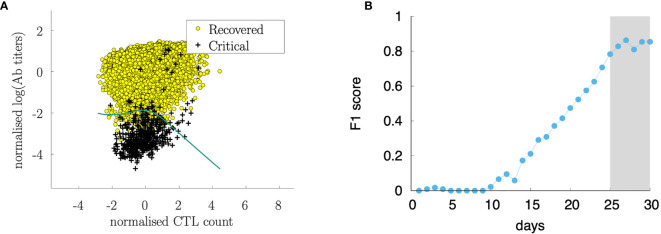
Sørensen-Dice coefficient (F1 score) of a logistic regression ML model to predict the outcome (recovered/critical) from *Tc(t)* and *Ab(t)* at various days **(B)**. The analysis shows that starting from day 25 from infection, the cytotoxic T cell counts and the antibody titers are informative for predicting the outcome. Panel **(A)** shows the discriminating curve of the measurements at day 25.

**Figure 14A** shows the features *x_i_* corresponding to the recovered cases (i.e., *y_i_* = 0) represented as yellow circles and the critical cases (i.e., *y_i_* = 1) corresponding to black daggers. This panel shows the best separation curve found after training a logistic regression model on the training sample *x_i_* = [*Tc*(25), *Ab*(25)], namely, Tc count and antibody titers measured at day 25 from infection.

**Figure 14A** shows the data set after the classification in recovered and critical and the separation curve. In the figure, the data set corresponds to the observation at day 30 while the analysis has been conducted at different time points. **Figure 14B** shows how the classification accuracy increases over time. In this panel, we plot the Sørensen-Dice coefficient [most known as the F1 score (76)] which increases when the assessment is made by using features (i.e., Tc and Ab measurements) later as the infection and corresponding immune response develops. Interestingly, before day 10 after the infection it is not possible to find a meaningful classification criterion that predicts the outcome, while the Sørensen-Dice coefficient increases to a high value already at day 25 indicating that 25 days after infection, the level of immune activation represented by the antibody titers and cytotoxic counts is predictive of the clinical outcome.

## Discussion and Conclusions

The immunological correlates of COVID-19 are far from being elucidated in clinical studies. Simulation studies can help to disentangle the importance of factors such as a reduced ability to mount an efficient (i.e., not off-target) immune response due to age or the infective viral load determining the initial viral burden.

We have set up a computational model that simulates the infection with a varying dosage of the virus and with a slightly different affinity to the ACE2 receptor of target cells, in individuals with different immunological competence.

The results of a large number of simulations that we call *virtual* or *in-silico cohort* demonstrate that the great variability observed in the real pandemic can be the mere result of such diversity in both viral and human characteristics.

The computational model used can explain several clinical observations of SARS-CoV-2 infection. In particular, it evidences the importance of the humoral response in discriminating efficiently from poor immune responses which fail to completely clear the infection and, in some cases, bring the viral load down below a threshold value while showing no markers of symptoms.

The model has been tuned for parameters able to reproduce the relationship of age with the disease severity (cf. **Figure 3**). Starting from that, any other observation revealed an emergent property of such a complex simulation environment. In particular, we observe the correlations among infective viral load *V*_0_ and severity, among immunological (in)competence (thus age) and severity, among the overall cytokine levels and symptoms (i.e., a virtual cytokine storm), and, finally, the key role of the humoral response in clearing the infection yet sustained by the cytotoxic activity (cf. **Figure 12**).

Indeed recent clinical data suggest that several hyper-inflammation markers can serve as accurate and reliable tools to identify mild or severe cases of COVID-19 infection (77).

Within cytokines, interleukin-6 (IL-6) is widely accepted to play a pivotal role, therefore it has been considered a possible therapeutic target. Indeed, the IL-6-receptor antagonist tocilizumab, has been used to treat patients with severe COVID-19 symptoms and pneumonia. However, clinical trials exploring tocilizumab’s therapeutic effects in patients with life-threatening SARS-CoV-2 infection have yielded very conflicting results (78). It should be noted that IL-6 production *in vivo* and real patients is usually accompanied by a relevant array of other cytokines and chemokines, and because of this, the therapeutic activity of tocilizumab might have been significantly hampered in some patients. There are also other potential confounding factors, for example, types of patients recruited in different trials and timing of treatment (early vs late).

It should be pointed out that in the context of this rapidly evolving scenario, defining best clinical practices is very challenging since results from ongoing trials are updated globally on a daily basis. Also in a global pandemic overall population outcomes may be measurable only during a long follow-up.

Our simulation study identifies day 25 after infection (which we can roughly associate to about day 15^th^-18^th^ after the appearance of the symptoms) as the time for predictive measurement of the antibody response to assess the risk of developing a severe form of the diseases. Before that time, our data suggest that the prediction is not statistically meaningful. Manifestly, the choice of antibody titers as marker to test for predictivity is due to the large availability of testing facilities and the short time and low cost for obtaining such readouts in clinics.

The model is restricted in many aspects. It simplifies reality and works with a limited number of mechanisms and a reduced diversity. For instance, it does not include type I IFN which has been recently evidenced as potentially important in the resolution of the infection (76, 79–81). However, type I IFN dynamics during SARS-CoV-2 infection are not yet fully clarified and future studies will explore whether IFN production is reduced at the beginning of infection or whether it is delayed or exhausted after an initial normal response (82–84).

Moreover, our model does not reproduce diverse organs and tissues and therefore we cannot observe site-specific pathological problems, including the spatial extension of pneumonia. Nevertheless, the analysis conducted in the present work accounts for such limitations and the results obtained can be reasonably considered independent of such restrictions.

Of course, there are some cases reported in the literature in which the course of infection has been extremely long, especially in severely immunocompromised patients. However, the very complex and lengthy dynamics taking place in those cases are beyond the scope of this study, which instead represents at large the majority of observed cases.

Finally, we should consider that the clinical ground of observation inevitably starts much later than in our model, as people ask for medical attention only after developing symptoms or after knowing of accidentally having been in contact with patients/carriers. Therefore, the window of observation we consider in this paper is recapitulating more precisely the infection dynamics of the early days.

Potential study directions should cover the duration of immunity, either natural or induced by the vaccine, including ways to indirectly verify it, the impact of immunosenescence on the elicited immunity, or the combined effect of monoclonal antibody and vaccines in potential future therapies.

Despite the extraordinary complexity of the immune system dynamics, the progress of simulation platforms suggests that a more intense interaction between clinicians and researchers in the computational model could bring these models to the desired quality for deployment in the medical field.

## Data Availability Statement

The original contributions presented in the study are included in the article/**Supplementary Material**. Further inquiries can be directed to the corresponding author.

## Author Contributions

FC and AL conceived the work. DD provided data. PL and AS provided useful insights and comments. All authors contributed to the writing of the manuscript. All authors contributed to the article and approved the submitted version.

## Funding

FC acknowledges partial funding from the European Union’s IMI 2 Joint Undertaking (JU) under grant agreement No. 853989 (project ERA4TB) and from the European Union’s Horizon 2020 research and innovation programme under grant agreement No 826121 (project iPC).

## Conflict of Interest

The authors declare that the research was conducted in the absence of any commercial or financial relationships that could be construed as a potential conflict of interest.

## Publisher’s Note

All claims expressed in this article are solely those of the authors and do not necessarily represent those of their affiliated organizations, or those of the publisher, the editors and the reviewers. Any product that may be evaluated in this article, or claim that may be made by its manufacturer, is not guaranteed or endorsed by the publisher.

## References

[1] World Health Organization. Available at: https://covid19.who.int.

[2] Johns Hopkins University. Available at: https://coronavirus.jhu.edu/data/mortality.

[3] Hu B Guo H Zhou P Shi Z-L. Characteristics of SARS-CoV-2 and COVID-19. Nat Rev Microbiol (2021) 19:141–54. 10.1038/s41579-020-00459-7 PMC7537588 33024307

[4] Adiga A Dubhashi D Lewis B Marathe M Venkatramanan S Vullikanti A. Mathematical Models for COVID-19 Pandemic: A Comparative Analysis. J Indian Inst Sci (2020) 100:793–807. 10.1007/s41745-020-00200-6 PMC7596173 33144763

[5] Mohamadou Y Halidou A Kapen PT. A Review of Mathematical Modeling, Artificial Intelligence and Datasets Used in the Study, Prediction and Management of COVID-19. Appl Intell (2020) 50:3913–25. 10.1007/s10489-020-01770-9 PMC7335662 34764546

[6] Castiglione F Celada F. Immune System Modeling and Simulation. Boca Raton: CRC Press (2015).

[7] Takaba H Takayanagi H. The Mechanisms of T Cell Selection in the Thymus. Trends Immunol (2017) 38(11):805–16. 10.1016/j.it.2017.07.010 28830733

[8] Castiglione F Santoni D Rapin N. “Ctls’ Repertoire Shaping in the Thymus: A Monte Carlo Simulation.,”. Autoimmunity (2011) 44(4):261–70. 10.3109/08916934.2011.523272 21244330

[9] Fekety FR.The Clonal Selection Theory of Acquired Immunity. Yale J Biol Med (1960) 32(6):480.

[10] Silverstein AM. The Clonal Selection Theory: What it Really Is and Why Modern Challenges are Misplaced. Nat Immunol (2002) 3(9):793–6. 10.1038/ni0902-793 12205463

[11] Lederberg J. Genes and Antibodies: Do Antigens Bear Instructions for Antibody Specificity or do They Select Cell Lines That Arise by Mutation? Science (1959) 129(3364):1649–53. 10.1126/science.129.3364.1649 13668512

[12] Brenner S Milstein C. Origin of Antibody Variation. Nature (1966) 211(5046):242–3. 10.3109/00365526809180138 5965537

[13] Tonegawa S. Somatic Generation of Antibody Diversity. Nature (1983) 302(5909):575–81. 10.1038/302575a0 6300689

[14] Papavasiliou FN Schatz DG. Somatic Hypermutation of Immunoglobulin Genes. Cell (2002) 109(2):S35–44. 10.1016/S0092-8674(02)00706-7 11983151

[15] Hayflick L Moorhead PS. The Serial Cultivation of Human Diploid Cell Strains. Exp Cell Res (1961) 25(3):585–621. 10.1016/0014-4827(61)90192-6 13905658

[16] Shay JW Wright WE. Hayflick, His Limit, and Cellular Ageing. Nat Rev Mol Cell Biol (2000) 1(1):72–6. 10.1038/35036093 11413492

[17] Schwartz RH. T Cell Anergy. Annu Rev Immunol (2003) 21(1):305–34. 10.1146/annurev.immunol.21.120601.141110 12471050

[18] Saibil SD Deenick EK Ohashi PS. The Sound of Silence: Modulating Anergy in T Lymphocytes. Curr Opin Immunol (2007) 19(6):658–64. 10.1016/j.coi.2007.08.005 17949964

[19] v. Nossal GJ Pike BL. Clonal Anergy: Persistence in Tolerant Mice of Antigen-Binding B Lymphocytes Incapable of Responding to Antigen or Mitogen. Proc Natl Acad Sci (1980) 77(3):1602–6. 10.1073/pnas.77.3.1602 PMC348545 6966401

[20] Yarkoni Y Getahun A Cambier JC. Molecular Underpinning of B-Cell Anergy. Immunol Rev (2010) 237(1):249–63. 10.1111/j.1600-065X.2010.00936.x PMC2968701 20727040

[21] Matzinger P. Tolerance, Danger, and the Extended Family. Annu Rev Immunol (1994) 12(1):991–1045. 10.1146/annurev.iy.12.040194.005015 8011301

[22] Gallucci S Matzinger P. Danger Signals: SOS to the Immune System. Curr Opin Immunol (2001) 13(1):114–9. 10.1016/S0952-7915(00)00191-6 11154927

[23] Pradeu T Cooper EL. The Danger Theory: 20 Years Later. Front Immunol (2012) 3:287:1–9. 10.3389/fimmu.2012.00287 23060876 PMC3443751

[24] Jerne NK. Towards a Network Theory of the Immune System. Ann Immunol (1974) 125C(1-2):373–89. 4142565

[25] Menshikov I Beduleva L Frolov M Abisheva N Khramova T Stolyarova E. The Idiotypic Network in the Regulation of Autoimmunity: Theoretical and Experimental Studies. J Theor Biol (2015) 375:32–9. 10.1016/j.jtbi.2014.10.003 25445185

[26] Castiglione F Poccia F D’Offizi G Bernaschi M. Mutation, Fitness, Viral Diversity, and Predictive Markers of Disease Progression in a Computational Model of HIV Type 1 Infection. AIDS Res Hum Retroviruses (2004) 20(12):1314–23. 10.1089/aid.2004.20.1314 15650424

[27] Castiglione F Duca K Jarrah A Laubenbacher R Hochberg D Thorley-Lawson D. Simulating Epstein-Barr Virus Infection With C-ImmSim. Bioinformatics (2007) 23(11):1371–7. 10.1093/bioinformatics/btm044 17341499

[28] Pappalardo F Lollini P-L Castiglione F Motta S. Modeling and Simulation of Cancer Immunoprevention Vaccine. Bioinformatics (2005) 21(12):2891–7. 10.1093/bioinformatics/bti426 15817697

[29] von Eichborn J Woelke AL Castiglione F Preissner R. VaccImm: Simulating Peptide Vaccination in Cancer Therapy. BMC Bioinformatics (2013) 14:1–9. 10.1186/1471-2105-14-127 23586423 PMC3651379

[30] Castiglione F Sleitser V Agur Z. Analysing Hypersensitivity to Chemotherapy in a Cellular Automata Model of the Immune System, London Chapman & Hall/CRC Press (UK) (2003) pp 333–365

[31] Prana V Tieri P Palumbo MC Mancini E Castiglione F. Modeling the Effect of High Calorie Diet on the Interplay Between Adipose Tissue, Inflammation, and Diabetes. Comput Math Methods Med (2019) 2019:1–8:2019. 10.1155/2019/7525834 PMC6378014 30863457

[32] Baldazzi V Paci P Bernaschi M Castiglione F. Modeling Lymphocyte Homing and Encounters in Lymph Nodes. BMC Bioinformatics (2009) 10:1–11. 10.1186/1471-2105-10-387 19939270 PMC2790470

[33] Castiglione F Tieri P Palma A Jarrah AS. Statistical Ensemble of Gene Regulatory Networks of Macrophage Differentiation. BMC Bioinformatics (2016) 17:119–28. 10.1186/s12859-016-1363-4 PMC5260144 28155642

[34] Castiglione F Ghersi D Celada F. Computer Modeling of Clonal Dominance: Memory-Anti-Naïve and Its Curbing by Attrition. Front Immunol (2019) 10:1513:1–12. 10.3389/fimmu.2019.01513 31338096 PMC6626922

[35] Madonia A Melchiorri C Bonamano S Marcelli M Bulfon C Castiglione F. Computational Modeling of Immune System of the Fish for a More Effective Vaccination in Aquaculture. Bioinformatics (2017) 33(19):3065–71. 10.1093/bioinformatics/btx341 28549079

[36] Kar T Narsaria U Basak S Deb D Castiglione F Mueller DM. A Candidate Multi-Epitope Vaccine Against SARS-CoV-2. Sci Rep (2020) 10(1):10895. 10.1038/s41598-020-67749-1 32616763 PMC7331818

[37] Abraham Peele K Srihansa T Krupanidhi S Ayyagari VS Venkateswarulu TC. Design of Multi-Epitope Vaccine Candidate Against SARS-CoV-2: A in-Silico Study. J Biomol Struct Dyn (2020) 39(10):3793–801. 10.1080/07391102.2020.1770127 PMC7284139 32419646

[38] Kohler B Puzone R Seiden PE Celada F. A Systematic Approach to Vaccine Complexity Using an Automaton Model of the Cellular and Humoral Immune System I. Viral Characteristics and Polarized Responses. Vaccine (2000) 19(7–8):862–76. 10.1016/S0264-410X(00)00225-5 11115710

[39] Rapin N Lund O Bernaschi M Castiglione F. Computational Immunology Meets Bioinformatics: The Use of Prediction Tools for Molecular Binding in the Simulation of the Immune System. PloS One (2010) 5(4):e9862. 10.1371/journal.pone.0009862 20419125 PMC2855701

[40] Lin HH Zhang GL Tongchusak S Reinherz EL Brusic V. Evaluation of MHC-II Peptide Binding Prediction Servers: Applications for Vaccine Research. BMC Bioinformatics (2008) 9(Suppl 12):S22:1–13. 10.1186/1471-2105-9-S12-S22 PMC2638162 19091022

[41] Lin HH Ray S Tongchusak S Reinherz EL Brusic V. Evaluation of MHC Class I Peptide Binding Prediction Servers: Applications for Vaccine Research. BMC Immunol (2008) 13:1–13. 10.1186/1471-2172-9-8 PMC2323361 18366636

[42] Lund O Nielsen M Kesmir C Petersen AG Lundegaard C Worning P. Definition of Supertypes for HLA Molecules Using Clustering of Specificity Matrices. Immunogenetics (2004) 55(12):797–810. 10.1007/s00251-004-0647-4 14963618

[43] Nielsen M Lundegaard C Worning P Hvid CS Lamberth K Buus S. Improved Prediction of MHC Class I and Class II Epitopes Using a Novel Gibbs Sampling Approach. Bioinformatics (2004) 20(9):1388–97. 10.1093/bioinformatics/bth100 14962912

[44] Nielsen M Lundegaard C Lund O. Prediction of MHC Class II Binding Affinity Using SMM-Align, a Novel Stabilization Matrix Alignment Method. BMC Bioinformatics (2007) 8(1):238. 10.1186/1471-2105-8-238 17608956 PMC1939856

[45] Allele Frequency Net Database. Available at: http://www.allelefrequencies.net.

[46] Gonzalez-Galarza FF McCabe A Melo dos Santos EJ Jones J Takeshita L Ortega-Rivera ND. Allele Frequency Net Database (AFND) 2020 Update: Gold-Standard Data Classification, Open Access Genotype Data and New Query Tools. Nucleic Acids Res (2019) 48(D1):D783–8. 10.1093/nar/gkz1029 PMC7145554 31722398

[47] IEDB Analysis Resource. Available at: http://tools.iedb.org/analyze/html/mhc_binding.html.

[48] Nielsen M Lundegaard C Worning P Lauemølle LS Lamberth K Buus S. Reliable Prediction of T-Cell Epitopes Using Neural Networks With Novel Sequence Representations. Protein Sci (2003), 12(5):1007–17. 10.1110/ps.0239403 PMC2323871 12717023

[49] Lundegaard C Lamberth K Harndahl M Buus S Lund O Nielsen M. NetMHC-3.0: Accurate Web Accessible Predictions of Human, Mouse and Monkey MHC Class I Affinities for Peptides of Length 8–11. Nucleic Acids Res (2008) 36(suppl_2):W509–12. 10.1093/nar/gkn202 PMC2447772 18463140

[50] Andreatta M Nielsen M. Gapped Sequence Alignment Using Artificial Neural Networks: Application to the MHC Class I System. Bioinformatics (2016) 32(4):511–7. 10.1093/bioinformatics/btv639 PMC6402319 26515819

[51] NetMHCIIpan 3.2 Server. Available at: www.cbs.dtu.dk/services/NetMHCIIpan.

[52] Jensen KK Andreatta M Marcatili P Buus S Greenbaum JA Yan Z. Improved Methods for Predicting Peptide Binding Affinity to MHC Class II Molecules. Immunology (2018) 154(3):394–406. 10.1111/imm.12889 29315598 PMC6002223

[53] de Bourcy CFA Angel CJL Vollmers C Dekker CL Davis MM Quake SR. Phylogenetic Analysis of the Human Antibody Repertoire Reveals Quantitative Signatures of Immune Senescence and Aging. Proc Natl Acad Sci (2017) 114(5):1105–10. 10.1073/pnas.1617959114 PMC5293037 28096374

[54] Pangrazzi L Weinberger B. T Cells, Aging and Senescence. Exp Gerontol (2020) 134:110887. 10.1016/j.exger.2020.110887 32092501

[55] Aiello A Farzaneh F Candore G Caruso C Davinelli S Gambino CM. Immunosenescence and Its Hallmarks: How to Oppose Aging Strategically? A Review of Potential Options for Therapeutic Intervention. Front Immunol (2019) 10:1–19. 10.3389/fimmu.2019.02247 31608061 PMC6773825

[56] Cline M Hutton JJ. Hematology and Oncology, Internal Medicine. Boston: Little, Brown (1983).

[57] Onder G Rezza G Brusaferro S. Case-Fatality Rate and Characteristics of Patients Dying in Relation to COVID-19 in Italy. JAMA (2020). 10.1001/jama.2020.4683 32203977

[58] Liu Z Bing X Zhi XZ. The Epidemiological Characteristics of an Outbreak of 2019 Novel Coronavirus Diseases (COVID-19) in China. Chin J Epidemiol (2020) 41(2):145–51. 10.3760/cma.j.issn.0254-6450.2020.02.003 32064853

[59] Wei Y Zeng W Huang X Li J Qiu X Li H. Clinical Characteristics of 276 Hospitalized Patients With Coronavirus Disease 2019 in Zengdu District, Hubei Province: A Single-Center Descriptive Study. BMC Infect Dis (2020) 20(1):549. 10.1186/s12879-020-05252-8 32727456 PMC7388483

[60] Zhao D Wang M Wang M Zhao Y Zheng Z Li X. Asymptomatic Infection by SARS-CoV-2 in Healthcare Workers: A Study in a Large Teaching Hospital in Wuhan, China. Int J Infect Dis (2020) 99:219–25. 10.1016/j.ijid.2020.07.082 PMC7836921 32758693

[61] Zhang S-Y Lian J-S Hu J-H Zhang X-L Lu Y-F Cai H. Clinical Characteristics of Different Subtypes and Risk Factors for the Severity of Illness in Patients With COVID-19 in Zhejiang, China. Infect Dis Poverty (2020) 9(1):85. 10.1186/s40249-020-00710-6 32641121 PMC7341711

[62] Verity R Okell LC Dorigatti I Winskill P Whittaker C Imai N. Estimates of the Severity of Coronavirus Disease 2019: A Model-Based Analysis. Lancet Infect Dis (2020) 20(6):669–77. 10.1016/S1473-3099(20)30243-7 PMC7158570 32240634

[63] Cascella M Rajnik M Cuomo A Dulebohn SC di Napoli R. Features, Evaluation, and Treatment of Coronavirus (COVID-19). Treasure Island (FL): StatPearls Publishing. (2021). 32150360

[64] Wiersinga WJ Rhodes A Cheng AC Peacock SJ Prescott HC. Pathophysiology, Transmission, Diagnosis, and Treatment of Coronavirus Disease 2019 (COVID-19). JAMA (2020) 324(8):782. 10.1001/jama.2020.12839 32648899

[65] Italian Ministry of Health Report. Available at: http://www.salute.gov.it/portale/nuovocoronavirus/dettaglioNotizieNuovoCoronavirus.jsp?lingua=italiano&menu=notizie&p=dalministero&id=4998.

[66] Westblade LF Brar G Pinheiro LC Paidoussis D Rajan M Martin P. SARS-CoV-2 Viral Load Predicts Mortality in Patients With and Without Cancer Who Are Hospitalized With COVID-19. Cancer Cell (2020) 38(5):661–71. 10.1016/j.ccell.2020.09.007 PMC7492074 32997958

[67] Petrilli CM Jones SA Yang J Rajagopalan H O'Donnell L Chernyak Y. Factors Associated With Hospital Admission and Critical Illness Among 5279 People With Coronavirus Disease 2019 in New York City: Prospective Cohort Study. BMJ (2020) 369:m1966. 10.1136/bmj.m1966. pp. 1–15. 32444366 PMC7243801

[68] Chen X Zhao B Qu Y Chen Y Xiong J Feng Y. Detectable Serum Severe Acute Respiratory Syndrome Coronavirus 2 Viral Load (RNAemia) Is Closely Correlated With Drastically Elevated Interleukin 6 Level in Critically Ill Patients With Coronavirus Disease 2019. Clin Infect Dis (2020) 71(8):1937–42. 10.1093/cid/ciaa449 PMC7184354 32301997

[69] Pedersen SF Ho Y-C. SARS-CoV-2: A Storm Is Raging. J Clin Invest (2020) 130(5):2202–5. 10.1172/JCI137647 PMC7190904 32217834

[70] Lucas C Wong C Klein C Castro C Silva C Sundaram C. Longitudinal Analyses Reveal Immunological Misfiring in Severe COVID-19. Nature (2020) 584(7821):463–9. 10.1038/s41586-020-2588-y PMC7477538 32717743

[71] Mueller AL McNamara MS Sinclair DA. Why Does COVID-19 Disproportionately Affect Older People? Aging (2020) 12(10):9959–81. 10.18632/aging.103344 PMC7288963 32470948

[72] Chen G Wu D Guo W Cao Y Huang D Wang H. Clinical and Immunological Features of Severe and Moderate Coronavirus Disease 2019,”. J Clin Invest (2020) 130(5):2620–9. 10.1172/JCI137244 PMC7190990 32217835

[73] Lynch KL Whitman JD Lacanienta NP Beckerdite EW Kastner SH Shy BR. Magnitude and Kinetics of Anti–Severe Acute Respiratory Syndrome Coronavirus 2 Antibody Responses and Their Relationship to Disease Severity. Clin Infect Dis (2020) 72(2):301–8. 10.1093/cid/ciaa979 PMC7454426 33501951

[74] Bastos M Tavaziva G Abidi SK Campbell JR Haraoui L-P Johnston JC. Diagnostic Accuracy of Serological Tests for Covid-19: Systematic Review and Meta-Analysis. BMJ (2020) 370(8256):m2516. 10.1136/bmj.m2516 32611558 PMC7327913

[75] Muecksch F Wise H Batchelor B Squires M Semple E Richardson C. Longitudinal Analysis of Clinical Serology Assay Performance and Neutralising Antibody Levels in COVID19 Convalescents. medRxiv (2020) 1–22. 10.1101/2020.08.05.20169128

[76] Dice LR. Measures of the Amount of Ecologic Association Between Species. Ecology (1945) 26(3):297–302. 10.2307/1932409

[77] Rowaiye AB Okpalefe OA Onuh Adejoke O Ogidigo JO Hannah Oladipo O Ogu AC. Attenuating the Effects of Novel COVID-19 (SARS-CoV-2) Infection-Induced Cytokine Storm and the Implications. J Inflamm Res (2021) 14:1487–510. 10.2147/JIR.S301784 PMC8057798 33889008

[78] Pelaia C Calabrese C Garofalo E Bruni A Vatrella A Pelaia G. Therapeutic Role of Tocilizumab in SARS-CoV-2-Induced Cytokine Storm: Rationale and Current Evidence. Int J Mol Sci (2021) 22(6):3059. 10.3390/ijms22063059 33802761 PMC8002419

[79] Ribero M Jouvenet N Dreux M Nisole S. Interplay Between SARS-CoV-2 and the Type I Interferon Response. PloS Pathog (2020) 16(7):e1008737. 10.1371/journal.ppat.1008737 32726355 PMC7390284

[80] Lei X Dong X Ma R Wang W Xiao X Tian Z. Activation and Evasion of Type I Interferon Responses by SARS-CoV-2. Nat Commun (2020) 11(1):1–12. 10.1038/s41467-020-17665-9 32733001 PMC7392898

[81] Bocharov G Casella V Argilaguet J Grebennikov D Güerri-Fernandez R Ludewig B. Numbers Game and Immune Geography as Determinants of Coronavirus Pathogenicity. Front Cell Infect Microbiol (2020) 10:1–4. 10.3389/fcimb.2020.559209 33194799 PMC7645103

[82] Hadjadj J Yatim N Barnabei L Corneau A Boussier J Smith N. Impaired Type I Interferon Activity and Inflammatory Responses In Severe COVID-19 Patients. Science (2020) 369(6504):718–24. 10.1126/science.abc6027 PMC7402632 32661059

[83] Zhang Q Bastard P Liu Z Le Pen J Moncada-Velez M Chen J. Inborn Errors of Type I IFN Immunity in Patients With Life-Threatening COVID-19. Science (2020) 370(6515):1–13. 10.1126/science.abd4570 PMC7857407 32972995

[84] Vieira JM Ricardo OMP Hannas CM Kanadani TCM Prata TDS Kanadani FN. What do We Know About COVID-19? A Review Article. Rev Assoc Med Bras (1992) 66(4):534–40. 10.1590/1806-9282.66.4.534 32578792

