# Supplementary material for: From Infection to Immunity: Understanding the Response to SARS-CoV2 Through In-Silico Modeling
Source: Front Immunol. 2021 Sep 7;12:646972. doi: 10.3389/fimmu.2021.646972 (PMC8453017; doi:10.3389/fimmu.2021.646972)
Supplement: Supplementary file 1 [file DataSheet_1.pdf]

Supplementary Material

Appendix A. The sequence of events from virus infection to immune response

The system is in a stable state (apart from random fluctuations due to natural cell death/birth of cells) until an antigen is injected. In this case the virus SARS-CoV-2 is applied at day 0. It follows a sequence of stochastic events promoting cells duplication, cytokine secretion and eventually culminating in the humoral and cellular immune response. Due to the high degree of details of the algorithms enacting such events, an agent-based model is not described by means of mathematical formulas but rather by *Rules* expressed in natural language without sacrificing rigor. The Rules of the agent-based automaton accounting for the main part of immune response to SARS-CoV-2 are listed below. Each rule corresponds to a more-or-less complex algorithm whose details are here neglected because less relevant to the purpose of the present article.

Secretion of cytokines by a cell is done in bulk (*quanta*) roughly corresponding to one pg/ml[a]. All cytokine releases per cell correspond to an amount of 10^2 *quanta*, thus 10^2 pg/ml. Cytokine secretion by a cell takes place in a time step of the simulation corresponding to 8 hours of real life, whenever the conditions are satisfied (e.g., mate encountering, proper local conditions, …); the duration of the secretion is therefore 8 hours but the event is repeated until the same conditions cease to be satisfied. Yet, to note, at each step the event is fulfilled with a certain probability that depends on the same conditions.

Also, antibody secretion by plasma B lymphocytes is represented as bulk secretion. Plasma cells secrete antibodies continuously for their whole lifetime.

Cell stimulations are discrete events modeled as Bernoulli events with parameter p which depends on the specific event. In recognition events as for instance when a T-cell receptor recognizes a viral peptide in the context of class I HLA, p represent the molecular affinity (as described in section 2.2 of the main manuscript) and that has been previously calculated with immune-bioinformatics prediction tools. Antigen digestion and presentation of viral peptides together with class I or II HLA molecules is also modeled as a Bernoulli event with parameter p previously estimated with immune-bioinformatics prediction tools.

Cells (but memory lymphocytes which live an order of magnitude longer) and molecules have a predefined life-span. In details, the half-life of B lymphocytes, Plasma B cells, CD4 T helpers, CD8

[a] Sullivan et al. (2000). Measurement of cytokine secretion, intracellular protein expression, and mRNA in resting and stimulated peripheral blood mononuclear cells. Clinical and diagnostic laboratory immunology, 7(6), 920–924

T cytotoxic and Macrophages is set to one week. Those of Dendritic cells and Natural killers, two weeks [b]. Immunoglobulins IgM have a half-life of 10 days. IgG1 21 days and IgG2 18 days [c].

The virus has a virtually infinite life-span. The same for Epithelial (virus target) cells and immunocomplexes which are eliminated by macrophage uptake.

Cytokines and signalling molecules in general (IL-2, Danger, IFNg, IL-12, IL-4, TGFb, TNFa, IL-10, IL-6, IFNb, IL-18, IL-23) are all modelled with a half-life of about 3 days [d].

1. Infection: An infection dose $V(0) = V_0$ is injected into the simulated volume representing 10 microliters discretised in $L_x \times L_y \times L_z = 10 \times 5 \times 5 = 250$ lattice points. As described in the manuscript the parameter V_0 has been taken in the range $[5 - 5 \cdot 10^5]$.
2. Endocytosis: the virus enters epithelial cells (EP). This happens at each time step t and within each lattice point x hosting both $V(t)$ viral particles and the epithelial cell, for each of them, with probability $1 - (1 - p_A)^{V(t)}$ where the probability p_A is the parameter described in the manuscripts which has been taken in the interval $[10^{-3} - 10^{-1}]$ and has units day^{-1}.
3. Biosynthesis: the viral RNA and viral proteins are made and assembled into new virions that are released by budding (exocytosis) from infected cells (SARS-CoV-2 follows a *lysogenic cycle*, that is, it does not kill the host). The number of viral particles budding from a single infected cell has been chosen about 10^3 per day[e]. At this stage, infected/injured EP
   - DAMPs release: release danger signal (D) (generally indicating interferon, cytokines, DAMPs = damage associated molecular patterns)
   - Inflammation: release IL-6
   - Endocytic presentation: process the viral proteins leading to their presentation on class I HLA molecules
4. B phagocytosis: B cells phagocyte, internalise, process and present viral peptides on class II HLA
5. Response to Danger:

---

[b] Sprent at al. (1994) Lymphocyte life-span and memory. Science 265(5177):1395-1400

c Amanna et al. (2010) Mechanisms that determine plasma cell lifespan and the duration of humoral immunity. Immunol Rev. 236:125–138

[d] Kindt, T., Goldsby, R., Osborne, B., Kuby, J. and Kuby, J., 2007. Kuby immunology. New York: W.H. Freeman.

[e] Gladnikoff et al. (2008) Directly Monitoring Individual Retrovirus Budding Events Using Atomic Force Microscopy. Biophysical Journal, 94(1): 320 – 326

- NK response: Natural killer cells (NKs) release IFNg upon bystander stimulation by danger
- M response: Macrophages (M) respond to danger (e.g., DAMPs) via TLR4 releasing TNFa and IL-6

6. M activation: macrophages become activated by IFNg (activated M have a greater phagocytic activity). This is modeled as a Bernoulli event with parameter $p = c \times e^{-(\frac{I_x}{I_E})^2}$, where $c = 0.9$, I_x is local concentration of IFNg (i.e., in lattice site x) and I_E is a parameter representing the efficiency of interferon in activating M.
7. Active M
   - M phagocytosis: M internalise, process and present viral peptides on class II HLA; in presence of IFNg they release IL-12; they also release TNFa
   - DC activation: M release TNFa which activate dendritic cells (DC)
8. DC phagocytosis & endocytosys: DC phagocyte, internalise, process and present viral peptides on class II HLA (exocytic pathway) but also on class I HLA (endocytic pathway)
9. Th activation: in presence of danger signal, resting T helper lymphocytes are activated by interaction with peptide-bound HLAs on professional antigen presenting cells (M and DC, mainly DC) surface by means of specific interaction with their T-cell receptors (TCR); if no danger is present, the Th cells becomes anergic upon interaction of its TCR with the HLApepide complex
10. Th stimulation by APCs: activated Th interacting with antigen presenting cells (M, DC)
    - Th duplication: start clone expansion; 50% of the daughter cells become memory cells
    - Th cells release IL-2
    - M release IL-6
    - Th1 release IFNg
    - Th2 release IL-4
    - release IL-12 in presence of high local concentration of IFNg
    - Treg release TGFb and IL-10
11. Th stimulation by B: activated Th interacting with B cells
    - B duplication: stimulate B cells to start clone expansion; 50% of the daughter cells become memory cells
    - Th duplication: start clone expansion; 50% of the daughter cells become memory cells
    - release IL-2, IL-12
    - Th1 release IFNg
    - Th2 release IL-4
    - Treg release TGFb and IL-10

12. Th differentiation: depending on the local concentration of IFNg, IL-10, IL-4, IL-6, IFNb, IL-12, IL-18, IL-2, TGFb and IL23, active T helper cells undergo class switch into Th1 and Th2. This complex process is modeled as a gene-regulatory-network as described in Santoni et al.[f]
13. B differentiation: B cells differentiate to antibody-secreting plasma B cells (PLB). 50% of duplicating B cells become PLBs. If the B lymphocyte is a memory cells then it generates 80% of PLBs.
14. Isotype switch: B cells perform immunoglobulin class switching, that is, change production of immunoglobulin from the isotype IgM to the isotype IgG. This is modeled as a Bernoulli event with parameter $p = \frac{(I_x)^3}{C^3+(I_x)^3}$ where C is an arbitrary constant and I_x is the local (i.e., in the lattice point) concentration of the interleukin IL-2.
15. Antibodies production: Plasma cells secrete antibodies at a rate of about 2 ng/day[g].
16. Humoral response: antibodies inhibit viral particles by opsonization; the result are the immuno-complexes that are eventually cleared by macrophages
17. Tc activation: in presence of IL-2, resting cytotoxic T cells (Tc) are activated by the interaction of their TCR with DC presenting on class I HLA the viral peptides but only in presence of IL-2
18. Tc duplication: activated Tc interact with infected EP cells presenting viral peptides on class I HLA molecule
    - Cytotoxic response: kill infected EP (this will further release danger signal)
    - Tc start duplication. 50% of the daughter cells become memory cells

---

[f] Santoni et al. (2008) Implementation of a regulatory gene network to simulate the TH1/2 differentiation in an agent-based model of hypersensitivity reactions. Bioinformatics 24 (11), 1374-1380

[g] Brinkmann et al. (1993) T cell-dependent differentiation of human B cells into IgM, IgG, IgA, or IgE plasma cells: high rate of antibody production by IgE plasma cells, but limited clonal expansion of IgE precursors. Cell Immunol. 152:323–32.

Appendix B. HLA class I peptide list

Each column reports the peptides relative to the allele indicated in the first row. Each entry of the table shows the peptide, the rank score and the relative amino acid position. The relative rank score is used to directly compute the probability to successful bind the peptide to the HLA molecule thus presenting the HLA-peptide complex to the cell surface.

A0201	A2402	B3501	B4002
FLAFVVFLL 5.26 20-28	NYMPYFFTL 6.81 2167-2175	FPFTIYSLL 19.41 9-17	AEWFLAYIL 7.64 2325-2333
SLVKPSFYV 9.11 50-58	YYTSNPTTF 11.58 1536-1544	MGYINVFAF 24.08 1-9	RELHLSWEV 9.9 5484-5492
VLLFLAFVV 21.72 17-25	VYMPASWVM 12.54 3653-3661	YINVFAFPF 26.67 3-11	AELAKNVSL 16.15 2618-2626
FLLVTLAIL 39.95 26-34	TYACWHHSI 13.91 6147-6155	VPFWITIAY 2.43 3136-3144	REFLTRNPA 17.56 5820-5828
SVLLFLAFV 44.91 16-24	TYASALWEI 17.94 4090-4098	LPSLATVAY 2.57 3641-3649	HEGKTFYVL 18.08 1613-1621
YIDIGNYTV 10.59 73-81	WSMATYYLF 22.74 900-908	FAVDAAKAY 3.29 4272-4280	QEYADVFHL 18.48 5266-5274
FLEYHDVRV 36.35 108-116	LYENAFLPF 30.99 3606-3614	FAIGLALYY 5.25 5614-5622	HEVLLAPLL 19.05 1141-1149
YVVDDPCPI 40.67 31-39	TYKPNTWCI 31.59 2002-2010	LVAEWFLAY 5.66 2323-2331	GEFKLASHM 19.24 912-920
HLVDFQVTI 28.82 3-11	YYRSLPGVF 33.09 3010-3018	NVLEGSVAY 5.78 2937-2945	YELQTPFEI 19.31 249-257
FLAHIQWMV 2.45 3122-3130	FFASFYYVW 42.38 2386-2394	LVYAADPAM 6.16 4764-4772	GEAANFCAL 19.34 1705-1713
FLLPSLATV 2.81 3639-3647	MYASAVVLL 43.63 3684-3692	FVSLAIDAY 6.89 5250-5258	YENAFLPFA 20.53 3607-3615
ILFTRFFYV 3.15 2332-2340	EWFLAYILF 44.08 2326-2334	MVMCGGSLY 6.96 5058-5066	LEMELTPVV 22.16 1012-1020
SMWALIISV 3.32 3732-3740	SYYSLLMPI 45.86 4628-4636	YPNASFDNF 7.22 1920-1928	FENKTTLPV 25.51 6494-6502
YLDAYNMMI 3.6 6418-6426	IYLYLTFYL 47.7 3108-3116	YPGQGLNGY 8.31 1329-1337	GEYSHVVAF 26.1 3072-3080
FLLNKEMYL 3.7 3183-3191	QYIKWPWYI 13.22 1208-1216	VAVKMFDAY 8.52 2586-2594	VELKHFFFA 32.03 4827-4835
TLMNVLTLV 3.75 3710-3718	VYSTGSNVF 19.05 635-643	HVGEIPVAY 9.36 110-118	KENSYTTTI 32.41 1869-1877
VLFSTVFPL 4.5 4707-4715	NYNYLYRLF 28.87 448-456	YVNTFSSTF 9.47 2594-2602	HEFCSQHTM 32.67 5201-5209
YLNTLTLAV 4.53 6850-6858	YFPLQSYGF 41.54 489-497	LVSDIDITF 10.85 1270-1278	EETGLLMPL 33.84 725-733
LLLDDFVEI 4.97 6748-6756	VYFLQSINF 47.43 112-120	MPYFFTLLL 11.75 2169-2177	KEILVTYNC 35.25 4535-4543

ALLADKFPV 4.99 6244-6252	LYLYALVYF 49.63 106-114	YIFFASFYY 12.11 2384-2392	VEYCPIFFI 38.62 3763-3771
TMADLVYAL 5 4515-4523	SYFIASFRL 27.75 94-102	YVMHANYIF 12.44 7019-7027	IELKFNPPA 41.96 1686-1694
YLATALLTL 5.07 1675-1683	YFIASFRLF 48.15 95-103	HSIGFDYVY 12.47 6153-6161	FELEDFIPM 42.48 6714-6722
KLIEYTDFA 5.37 2901-2909		QVVDMSMTY 12.59 1582-1590	TERLKLFAA 45.15 5450-5458
MMISAGFSL 5.47 6424-6432		TPAFDKSAF 12.87 6352-6360	RELKVTFFP 46.3 1953-1961
VLWAHGFEL 5.78 6108-6116		LPGVYSVIY 13.23 3101-3109	YENFNQHEV 47.17 1135-1143
NLIDSYFVV 5.93 4456-4464		VPWDTIANY 13.33 2133-2141	HEHEIAWYT 48.55 234-242
WMVMFTPLV 6.06 3128-3136		MSNLGMPSY 13.57 2254-2262	FEYVSQPFL 16.77 168-176
WLMWLIINL 6.6 2363-2371		TVLCLTPVY 13.78 3090-3098	SEFRVYSSA 25.84 155-163
KLSYGIATV 6.77 5469-5477		LPVNVAFEL 14.41 6500-6508	FELLHAPAT 41.23 515-523
YMPYFFTLL 7.12 2168-2176		VPFVVSTGY 14.57 4730-4738	SELVIGAVI 43.47 136-144
LLFLMSFTV 7.19 3083-3091		FPLTSFGPL 14.72 4713-4721	
YTMADLVYA 7.34 4514-4522		FVVEVVDKY 14.91 4863-4871	
YLTNDVSFL 7.42 3115-3123		LPLTQYNRY 16.09 3199-3207	
FLARGIVFM 7.71 3753-3761		FSAVGNICY 16.33 2889-2897	
ALWEIQQVV 7.85 4094-4102		MADQAMTQM 16.41 4004-4012	
FVNEFYAYL 8.24 5132-5140		WVYKQFDTY 18.56 6433-6441	
ILHCANFNV 8.64 4699-4707		LPFKLTCAT 18.64 2738-2746	
AIFYLITPV 8.72 2785-2793		LPSYAAFAT 19.53 3951-3959	
KLNVGDYFV 9.02 5541-5549		IAMSAFAMM 19.56 3619-3627	
LVLSVNPYV 9.08 5364-5372		LAKDTTEAF 19.63 3900-3908	
FLNRFTTTL 9.14 3482-3490		NPHLMGWDY 19.87 5003-5011	
NLSDRVVFV 9.53 6100-6108		NAAISDYDY 21.38 4839-4847	
LMIERFVSL 9.68 5245-5253		QAWQPGVAM 21.65 6800-6808	
QLFFSYFAV 10.01 2348-2356		FCLEASFNY 22.36 2209-2217	

RIMTWLDMV 10.03 3662-3670		LASHMYCSF 22.93 916-924	
LLSAGIFGA 10.09 1148-1156		VVYRGTTTY 23.66 5532-5540	
LLLTILTSL 10.2 3583-3591		FSSTFNVPM 24.37 2598-2606	
FVDGVPFVV 10.2 4726-4734		NPPALQDAY 25.39 1691-1699	
LLADKFPVL 11.11 6245-6253		KPVETSNSF 25.51 2017-2025	
FLPRVFSAV 11.73 2884-2892		HVASCDAIM 26.09 6192-6200	
FVAAIFYLI 11.96 2782-2790		VVIPDYNTY 26.32 4072-4080	
YLASGGQPI 11.96 4283-4291		FVSDADSTL 27 6900-6908	
VMVELVAEL 12.28 84-92		MGIIAMSAF 27.34 3616-3624	
ILTSLLVLV 12.61 3587-3595		YVFCTVNAL 27.98 5678-5686	
LMWLIINLV 13.29 2364-2372		FVLTSHTVM 31.42 5548-5556	
VLAWLYAAV 13.4 3467-3475		FVVSTGYHF 34.54 4732-4740	
VLSFCAFAV 13.64 4266-4274		NALDQAISM 35.01 3725-3733	
LLMPILTLT 14.16 4632-4640		LATNNLVVM 35.31 590-598	
RLIDAMMFT 14.25 579-587		LIISVTSNY 37.11 3736-3744	
VMCGGSLYV 14.27 5059-5067		LPFAMGIIA 38.16 3612-3620	
GLNDNLLEI 14.57 445-453		LMNVLTLVY 38.72 3711-3719	
FLGRYMSAL 14.93 1642-1650		TVAYFNMVY 39.68 3646-3654	
KLMPVCVET 15.05 1387-1395		FAWWTAFVT 39.78 6984-6992	
FVMMSAPPA 15.07 1804-1812		MVTNNTFTL 41.13 807-815	
YVWKSYVHV 15.2 2392-2400		YGQQFGPTY 44.09 1590-1598	
TQWSLFFFL 15.7 3598-3606		FVNLKQLPF 44.48 6360-6368	
MLDMYSVML 15.97 5290-5298		LAVFDKNLY 45.24 1175-1183	
YLNSTNVTI 17.19 2270-2278		YVLGLAAIM 45.87 2339-2347	
TLIGDCATV 17.61 6907-6915		VVVNAANVY 45.93 1056-1064	

KLWAQCVQL 17.72 3886-3894		CTDDNALAY 46.96 4163-4171	
NLLKDCPAV 18.8 4480-4488		YLVQQESPF 47.27 1796-1804	
QLMCQPILL 18.97 2563-2571		DASGKPVPY 48.47 2924-2932	
IIWFLLLSV 19.01 2230-2238		LAAIMQLFF 49.25 2343-2351	
LLTNMFTPL 19.31 3026-3034		LALYNKYKY 49.28 3208-3216	
QMAPISAMV 19.63 2373-2381		IPFAMQMAY 2.99 896-904	
FLNGSCGSV 19.67 3403-3411		FAMQMAYRF 5.03 898-906	
SLAIDAYPL 20.82 5252-5260		LGAENSVAY 6.31 699-707	
FLMSFTVLC 23.05 3085-3093		SANNCTFEY 7.78 162-170	
MQLFFSYFA 23.12 2347-2355		WPWYIWLGF 8.91 1212-1220	
ILGTVSWNL 23.63 1367-1375		VASQSIIAY 8.93 687-695	
LLDDFVEII 23.67 6749-6757		LPFNDGVYF 9.15 84-92	
KLKDCVMYA 23.81 3678-3686		FVSNGTHWF 17.28 1095-1103	
SLPGVFCGV 24.07 3013-3021		LPPLLTDEM 25.98 861-869	
TLGVYDYLV 24.56 3807-3815		LPPAYTNSF 26.5 24-32	
WLPTGTLLV 24.8 6885-6893		AALQIPFAM 26.99 892-900	
SLLSVLLSM 25.87 3913-3921		FVFKNIDGY 28.18 192-200	
TLSEQLDFI 26.42 214-222		MIAQYTSAL 28.51 869-877	
ILLLDQALV 27.11 2569-2577		NATRFASVY 32.3 343-351	
LQLGFSTGV 27.69 6031-6039		FPREGVFVS 35.75 1089-1097	
AIMTRCLAV 28.95 6198-6206		YSSANNCTF 38.72 160-168	
MLWCKDGHV 29.26 6781-6789		LPFFSNVTW 39.55 56-64	
FLALCADSI 29.8 685-693		CVADYSVLY 40.54 361-369	
KMFDAYVNT 30.2 2589-2597		CPDGVKHVY 25.7 67-75	
SLIYSTAAL 30.84 2242-2250		LATCELYHY 30.47 12-20	

VLLSVLQQL 32.87 3871-3879		IPIQASLPF 3.4 35-43	
ILSPLYAFA 33.74 529-537		VAAGLEAPF 21.19 97-105	
FMCVEYCPI 33.94 3760-3768		FLCWHTNCY 41.02 146-154	
FTVLCLTPV 34.4 3089-3097		YANRNRFLY 16.49 39-47	
AMQTMLFTM 34.53 4028-4036		VATSRTLSY 31.18 170-178	
MMILSDDAV 35.14 5146-5154		FAYANRNRF 38.08 37-45	
YLITPVHVM 35.35 2788-2796		TPSGTWLTY 4.73 325-333	
KLNEEIAII 36.42 468-476		LPAADLDDF 10.96 395-403	
KLVNKFLAL 36.64 680-688		LPNNTASWF 14.87 45-53	
VMAYITGGV 37.8 597-605		FAPSASAFF 29.99 307-315	
MLFTMLRKL 37.82 4032-4040		KAYNVTQAF 32.16 266-274	
GLALYYPSA 37.83 5617-5625			
TLVPQEHYV 38.21 5562-5570			
SLENVAFNV 38.77 6452-6460			
KMVSLLSVL 39.75 3910-3918			
SQLGGLHLL 40.62 6694-6702			
VLLAPLLSA 40.78 1143-1151			
YLYLTFYLT 41.51 3109-3117			
NMLRIMASL 41.52 5020-5028			
FLRDGWEIV 42.13 641-649			
FLKKDAPYI 42.2 1278-1286			
NTFSSTFNV 43.65 2596-2604			
FLPGVYSVI 43.7 3100-3108			
MLSDTLKNL 44.66 6093-6101			
GLFKDCSKV 45.87 5930-5938			

KLNIKLLGV 46.87 3839-3847<br><br>TLGVLVPHV 48.05 103-111<br><br>YVFCTVNAL 48.78 5678-5686<br><br>SLPSYAAFA 48.84 3950-3958<br><br>YLQPRTFLL 5.36 269-277<br><br>FQFCNDPFL 9.18 133-141<br><br>FIAGLIAIV 10.29 1220-1228<br><br>SIIAYTMSL 13.54 691-699<br><br>RLQSLQTYV 16.66 1000-1008<br><br>FTISVTTEI 25.37 718-726<br><br>LLFNKVTLA 25.41 821-829<br><br>HLMSFPQSA 26.36 1048-1056<br><br>VLNDILSRL 33.57 976-984<br><br>KIADYNYKL 36.12 417-425<br><br>VVFLHVTYV 36.56 1060-1068<br><br>RLDKVEAEV 38.95 983-991<br><br>FVFLVLLPL 41.68 2-10<br><br>KLFIRQEEV 31.81 85-93<br><br>YLYALVYFL 2.67 107-115<br><br>LLYDANYFL 3.06 139-147<br><br>ALSKGVHFV 7.25 72-80<br><br>ALLAVFHSA 17.03 51-59<br><br>TVYSHLLLV 28.4 89-97<br><br>ALVYFLQSI 37 110-118<br><br>GLMWLSYFI 3.87 89-97			

KLLEQWNLV 7.57 15-23<br>FVLAAVYRI 16.52 65-73<br>SMWSFNPET 19.09 108-116<br>FLFLTWICL 32.26 26-34<br>FLWLLWPVT 32.54 53-61<br>TLACFVLAA 33.11 61-69<br>FIASFRLFA 46.41 96-104<br>LLLDRLNQL 14.81 222-230			

Appendix C. HLA class II peptides

Each column reports the peptides relative to the HLA indicated in the first row. Each entry of the table shows the peptide and the relative rank score. The relative rank score is used to directly compute the probability to successful bind the peptide to the HLA molecule thus presenting the HLA-peptide complex to the cell surface.

**DRB1_0701**	**DRB1_1501**
PFTIYSLLL 1.70	LLLCRMNSR 1.20
YVYSRVKNL 0.25	IYSLLLCRM 1.40
LAILTALRL 0.40	LAILTALRL 0.20
FYVYSRVKN 0.70	FYVYSRVKN 0.50
TLAILTALR 1.90	TLAILTALR 1.00
IITTVAAFH 1.10	YVYSRVKNL 1.90
LIIMRTFKV 0.04	IHFYSKWYI 0.40
FQVTIAEIL 0.25	IITTVAAFH 0.60
IIKNLSKSL 1.10	LIIMRTFKV 0.01
YIINLIIKN 1.80	LLIIMRTFK 0.17

FYLITPVHV 0.04	YIINLIIKN 1.00
YFVLTSHTV 0.06	IIKNLSKSL 1.30
FKHLIPLMY 0.08	INLIIKNLS 1.40
FRYMNSQGL 0.12	FHLYLQYIR 0.02
FTRSTNSRI 0.12	AYYFMRFRR 0.02
FSASTSAFV 0.12	FYAYLRKHF 0.03
AYYFMRFRR 0.17	FLAYILFTR 0.05
FVVSTGYHF 0.20	YLQYIRKLH 0.06
FVKHKHAFL 0.20	FMRFRRAFG 0.06
ARYMRSLKV 0.20	YYFMRFRRA 0.09
FHLYLQYIR 0.20	CLLNRYFRL 0.12
VRSIFSRTL 0.25	FLHFLPRVF 0.12
CLLNRYFRL 0.25	FCLLNRYFR 0.15
FLAHIQWMV 0.25	IFYLITPVH 0.15
LRKHFSMMI 0.30	LAYYFMRFR 0.17
FYAYLRKHF 0.30	FVKHKHAFL 0.20
FLALCADSI 0.30	FFKLVNKFL 0.25
LRANSAVKL 0.40	MMCYKRNRA 0.25
YYFMRFRRA 0.40	LYLQYIRKL 0.25
FLLNKEMYL 0.40	IIQFPNTYL 0.25
FLHFLPRVF 0.40	FKHLIPLMY 0.25
YRGTTTYKL 0.40	YWFFSNYLK 0.30
FFKLVNKFL 0.40	YLALYNKYK 0.30
YLNTLTLAV 0.40	IFFITGNTL 0.30
ICYTPSKLI 0.40	YYVWKSYVH 0.30

FNYLKSPNF 0.40	IMASLVLAR 0.30
YVMHANYIF 0.40	YNRYLALYN 0.30
YFYTSKTTV 0.40	FLAHIQWMV 0.30
LRLIDAMMF 0.40	WFFSNYLKR 0.30
IYSTAALGV 0.40	ILAYCNKTV 0.30
YRVTKNSKV 0.40	YFMRFRRAF 0.40
IFFITGNTL 0.50	FYLITPVHV 0.40
WFFSNYLKR 0.50	LALYYPSAR 0.40
YFVVKRHTF 0.50	KVKYLYFIK 0.40
LRIMASLVL 0.50	VMYMGTLSY 0.40
FCSQHTMLV 0.50	FYYVWKSYV 0.40
YDKLVSSFL 0.50	TMLFTMLRK 0.50
FAVSKGFFK 0.50	FYWFFSNYL 0.50
FFITGNTLQ 0.60	LRIMASLVL 0.50
YLQYIRKLH 0.60	FFLYENAFL 0.50
YWFFSNYLK 0.60	LILMTARTV 0.50
FKLTCATTR 0.60	IVKFISTCA 0.50
YLKLRSDVL 0.60	VFHLYLQYI 0.50
FKLVNKFLA 0.60	LLQLCTFTR 0.60
YCALAPNMM 0.70	FKLVNKFLA 0.60
YRRLISMMG 0.70	VNEFYAYLR 0.60
LSVLQQLRV 0.70	FLLNKEMYL 0.60
FVNLDNLRA 0.70	ISAMVRMYI 0.60
FLAYILFTR 0.70	FYFYTSKTT 0.60
LKLFAAETL 0.80	LRLIDAMMF 0.60

YYVWKSYVH 0.80	LHLLIGLAK 0.60
FYFYTSKTT 0.80	IICISTKHF 0.60
YIICISTKH 0.80	ARYMRSLKV 0.60
YMRSLKVPA 0.80	YFVLTSHTV 0.60
YVRNLQHRL 0.80	VLYYQNNVF 0.60
IIQFPNTYL 0.80	ILSLLSKGR 0.70
WNVVRIKIV 0.80	LFLLPSLAT 0.70
IERFVSLAI 0.80	IERFVSLAI 0.70
FYYVWKSYV 0.80	LRKHFSMMI 0.70
NYVFTGYRV 0.80	FAVSKGFFK 0.70
LVLSVNPYV 0.80	NYVFTGYRV 0.70
LKTLATHGL 0.90	MLRIMASLV 0.70
LFAAETLKA 0.90	LVLSVNPYV 0.70
IFYLITPVH 0.90	LKLFDRYFK 0.70
VHFISNSWL 0.90	IKNFKSVLY 0.70
YLNSTNVTI 0.90	FRYMNSQGL 0.70
SLSHRFYRL 0.90	VRSIFSRTL 0.70
IICISTKHF 0.90	YIICISTKH 0.70
TYFTQSRNL 1.00	YFVVKRHTF 0.80
HVISTSHKL 1.00	VHFISNSWL 0.80
IMASLVLAR 1.00	FVVSTGYHF 0.80
FNSVCRLMK 1.00	LISMMGFKM 0.80
FAYTKRNVI 1.00	AMMFVKHKH 0.80
FYWFFSNYL 1.00	FAMMFVKHK 0.80
FSYFAVHFI 1.00	FFSNYLKRR 0.80

VKILNNLGV 1.10	YLYFIKGLN 0.80
YFIKGLNNL 1.10	LSVLQQLRV 0.80
FMRFRRAFG 1.10	LMPILTLTR 0.80
YYRSLPGVF 1.10	ILRVYANLG 0.80
YVLMDGSII 1.10	LLILMTART 0.80
YLITPVHVM 1.10	MMFVKHKHA 0.80
MLRIMASLV 1.10	VLLILMTAR 0.90
LLKSIAATR 1.10	IAIILASFS 0.90
LILMTARTV 1.10	LYKMQRMLL 0.90
IQLSSYSLF 1.10	LHFLPRVFS 0.90
YLDAYNMMI 1.10	LYFIKGLNN 1.00
FCLLNRYFR 1.10	YKVYYGNAL 1.00
HFISNSWLM 1.10	IIAMSAFAM 1.00
ILAYCNKTV 1.10	FSNYLKRRV 1.00
FYILPSIIS 1.10	YMRSLKVPA 1.00
KFLTENLLL 1.10	LFAYTKRNV 1.00
IISVTSNYS 1.10	FKMFYKGVI 1.00
FFLYENAFL 1.20	LLIGLAKRF 1.10
YKVYYGNAL 1.20	FGLFCLLNR 1.10
FVFPLNSII 1.20	MVYMPASWV 1.10
ALYYPSARI 1.20	LNRYFRLTL 1.10
IKNFKSVLY 1.20	YVMHANYIF 1.10
PIQLSSYSL 1.30	LYVNKHAFH 1.10
LYLQYIRKL 1.30	YRRLISMMG 1.10
YNRYLALYN 1.30	YNLWNTFTR 1.20

FYLTNDVSF 1.30	YVRITGLYP 1.20
NYMLTYNKV 1.30	LYYQNNVFM 1.20
LREVRTIKV 1.30	YKHYTPSFK 1.20
FFSNYLKRR 1.30	YRLYLDAYN 1.20
SLLSKGRLI 1.30	LSTFISAAR 1.20
CQYLNTLTL 1.30	ILTLTRALT 1.20
LRVESSSKL 1.30	CLAYYFMRF 1.20
LIYSTAALG 1.30	FYILPSIIS 1.20
YKRNRATRV 1.40	IIFLEGETL 1.20
IQITISSFK 1.40	LKLFAAETL 1.30
YFTQSRNLQ 1.40	YIKWDLLKY 1.30
FYTSKTTVA 1.40	ILLLDQALV 1.30
FKLSYGIAT 1.40	FSYFAVHFI 1.30
YYRYNLPTM 1.40	IISVTSNYS 1.30
VCGVSAARL 1.40	LIINLVQMA 1.30
FSNYLKRRV 1.40	VKILNNLGV 1.40
FTTVDNINL 1.40	AIRHVRAWI 1.40
FISTCACEI 1.40	FVNLDNLRA 1.40
FCLEASFNY 1.50	VVQLTSQWL 1.40
YFMRFRRAF 1.50	ISQYSLRLI 1.40
LFYSYATHS 1.60	IQLLKSAYE 1.40
IVKFISTCA 1.60	TFFKLVNKF 1.40
LTLTRALTA 1.60	TCLAYYFMR 1.50
FIKGLNNLN 1.60	LTSMKYFVK 1.50
YVRITGLYP 1.60	ALYYPSARI 1.50

FYRLANECA 1.60	YVRNLQHRL 1.60
IPLTTAAKL 1.60	SLSHRFYRL 1.60
YVDNSSLTI 1.60	CVSFCYMHH 1.60
INIVGDFKL 1.60	VRRSFYVYA 1.60
TRVLSNLNL 1.70	IIKTIQPRV 1.60
LKLTDNVYI 1.70	YYRYNLPTM 1.70
ISQYSLRLI 1.70	LFCLLNRYF 1.70
YFNSVCRLM 1.70	VYSFLPGVY 1.70
MMCYKRNRA 1.70	VNSFSGYLK 1.70
YMSALNHTK 1.70	LVASIKNFK 1.70
HMVVKAALL 1.70	LLKSIAATR 1.70
KQLIKVTLV 1.70	FNSVCRLMK 1.70
LSTFISAAR 1.70	WMVMFTPLV 1.70
LSVVNARLR 1.80	VAYRKVLLR 1.70
LLQLCTFTR 1.80	YFIKGLNNL 1.80
GISQYSLRL 1.80	LLTILTSLL 1.80
FSSTFNVPM 1.80	IQITISSFK 1.80
LIINLVQMA 1.80	IYLYLTFYL 1.80
VMYMGTLSY 1.80	FLALCADSI 1.80
LRGTAVMSL 1.90	LTSFGPLVR 1.80
VRETMSYLF 1.90	FVMMSAPPA 1.80
HFIETISLA 1.90	LSLLSKGRL 1.90
VRIKIVQML 1.90	LALYNKYKY 1.90
MHAASGNLL 1.90	RYLALYNKY 1.90
YLAVFDKNL 1.90	FFSYFAVHF 1.90

FKLNEEIAI 1.90	IVQMLSDTL 1.90
IYQTSNFRV 0.06	MMGFKMNYQ 1.90
FQTLLALHR 0.08	VNKFLALCA 1.90
FAMQMAYRF 0.12	FQTLLALHR 0.09
FASVYAWNR 0.20	NYLYRLFRK 0.17
YLQPRTFLL 0.40	FAMQMAYRF 0.20
YRLFRKSNL 0.50	ITRFQTLLA 0.20
IAQYTSALL 0.60	YNYLYRLFR 0.25
FNATRFASV 0.60	IYQTSNFRV 0.40
ITRFQTLLA 0.60	FASVYAWNR 0.40
FTISVTTEI 0.70	YRLFRKSNL 0.50
FCTQLNRAL 0.80	LLALHRSYL 0.60
RFQTLLALH 1.10	IAQYTSALL 0.70
FFSNVTWFH 1.10	VYAWNRKRI 0.80
NYLYRLFRK 1.20	IIAYTMSLG 1.00
LALHRSYLT 1.20	FFSNVTWFH 1.00
LLFNKVTLA 1.40	YLYRLFRKS 1.10
VFRSSVLHS 1.50	LLFNKVTLA 1.30
YNYLYRLFR 1.50	RFQTLLALH 1.30
FGAGAALQI 1.70	VTWFHAIHV 1.30
FGAISSVLN 1.80	VLSFELLHA 1.60
IDRLITGRL 1.80	LALHRSYLT 1.70
FRVQPTESI 1.90	ITLCFTLKR 1.40
INITRFQTL 1.90	VYQLRARSV 1.60
FRVYSSANN 1.90	VKHVYQLRA 1.90

CVLGQSKRV 1.90	FLQSINFVR 0.30
VYQLRARSV 0.50	VTFFIYNKI 0.30
FVTVYSHLL 0.06	INFVRIIMR 0.40
FLQSINFVR 0.17	FVTVYSHLL 0.60
YNSVTSSIV 0.20	YFLQSINFV 0.70
VFHSASKII 0.40	VYFLQSINF 0.80
YYQLYSTQL 0.60	LVYFLQSIN 0.80
YFLQSINFV 0.80	LFVTVYSHL 1.00
INFVRIIMR 0.90	LLFVTVYSH 1.10
YQLYSTQLS 0.90	VRIIMRLWL 1.30
YFTSDYYQL 1.10	TFFIYNKIV 1.50
WQLALSKGV 1.10	NFVRIIMRL 1.60
LALSKGVHF 1.10	FMRIFTIGT 1.80
FVRATATIP 1.20	LSYFIASFR 0.03
VRATATIPI 1.40	FAAYSRYRI 0.08
LFVTVYSHL 1.40	FRLFARTRS 0.10
VYFLQSINF 1.70	SYFIASFRL 0.15
SYFIASFRL 0.05	FIASFRLFA 0.17
FVLAAVYRI 0.05	IASFRLFAR 0.20
FAAYSRYRI 0.08	FVLAAVYRI 0.25
FRLFARTRS 0.15	LLQFAYANR 0.40
LSYFIASFR 0.17	VILRGHLRI 1.20
YKLGASQRV 0.60	CFVLAAVYR 1.50
ITVATSRTL 0.80	RFLYIIKLI 1.70
IASFRLFAR 0.90	YYRRATRRI 1.50

FIASFRLFA 1.00<br>LLQFAYANR 1.40<br>LTYTGAIKL 0.20<br>YYRRATRRI 0.20<br>YRRATRRIR 1.20	QVILLNKHI 1.70

Appendix D. Example readout of a simulation

Figure 1 shows in panel A how the viral load $V(t)$ (both soluble, meaning outside infected cells, and proviral, meaning inside infected cells, and the sum of the two), varies with time: the viral particles of SARS-Cov-2 injected at day 0, peaking at about day 5 and start to decline after that and in correspondence to the appearance of antibody producing plasma cells (panel B of Figure 2). In the same panel the immunoglobulins titers are plotted (split in IgM and IgG, further split in IgG1 and IgG2) together with the immunocomplexes (IC) that are antibodies bound to viruses (i.e., opsonised viruses). This plot shows a humoral response surging at about day 12 and clearing the virus in about five days. It also shows that the antibody levels remain high for some time after the simulation ends at day 30.

Panel (B) plots in arbitrary scale the cytokine concentrations for interleukins, interferon and danger signal (inset plot of the same panel). In particular this run shows a high level of IFNg, IL-6, IL-2 and IL-12 elicited by the infection besides a moderate production of danger signal (D, in the inset plot of panel B). Release of cytokines follows from the dynamical rules characterizing the agent's behavior reported in Appendix A.

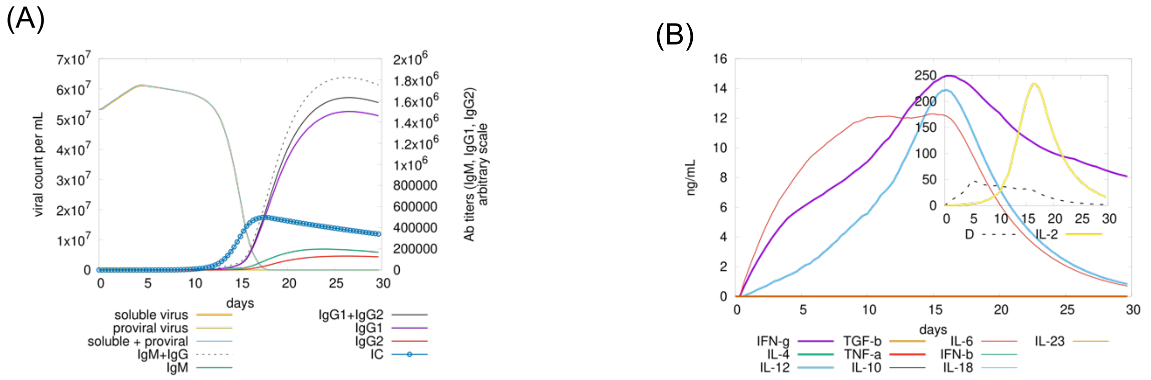

Figure 1 Viral load $V(t)$, immunoglobulins (IgM and IgG) and the immunocomplexes (IC) are shown in panel A. The same panel shows the viral particles of SARS-Cov-2 injected at day 0, peaking at about day 5 and start to decline after that and in correspondence to the appearance

of antibody producing plasma cells (panel B of Figure 2). Panel B shows the cytokines generated during the immune response (see text for details).

Panel A of Figure 2 shows the total counts of the B-cells in all phenotypes, that is, memory and not memory, and the three isotypes IgM, IgG1 and IgG2 (i.e., cells that will become plasma B cells producing IgM or IgG antibodies).

Panel B shows the count of antibody-generating plasma cells subdivided in the three classes IgM, IgG1 and IgG2.

Panel C gets into the details of the ABM simulation model by showing the counts of B-cells subdivided according to the internal state assumed. Worth to note that the cells enter the duplication state only after day 10 and until day 20 because besides presenting the viral peptides on their HLA molecules, they need to be stimulated by stimulated cognate helper T cells bearing the “correct” cell receptor (panel D of same Figure 2). Note also that upon clearance of the virus (cf. panel A of Figure 1) the B-cell population switches back to the “active” state and terminates the presentation of the viral proteins.

Panel D shows cell counts for CD4 T-cell population (total number, memory cells, not memory). Some days after the infection and upon successful interaction with antigen presenting cells, T helper lymphocytes start to duplicate and differentiate into memory. They also foster cytotoxicity (cf. panel A and B of Figure 3) and humoral response (cf. panel B of Figure 2) through secretion of cytokines (panel B of Figure 1). As for B cells in panel A, panel E shows Th counts specifying the internal state of the lymphocyte thus revealing the “activation” phase starting quite soon before day 5 and the duplication phase starting immediately after and ending at about day 20. Of note, some cell become anergic around day 15 for lack of “danger” (second) signal upon activation (Rule n.9 in Appendix A).

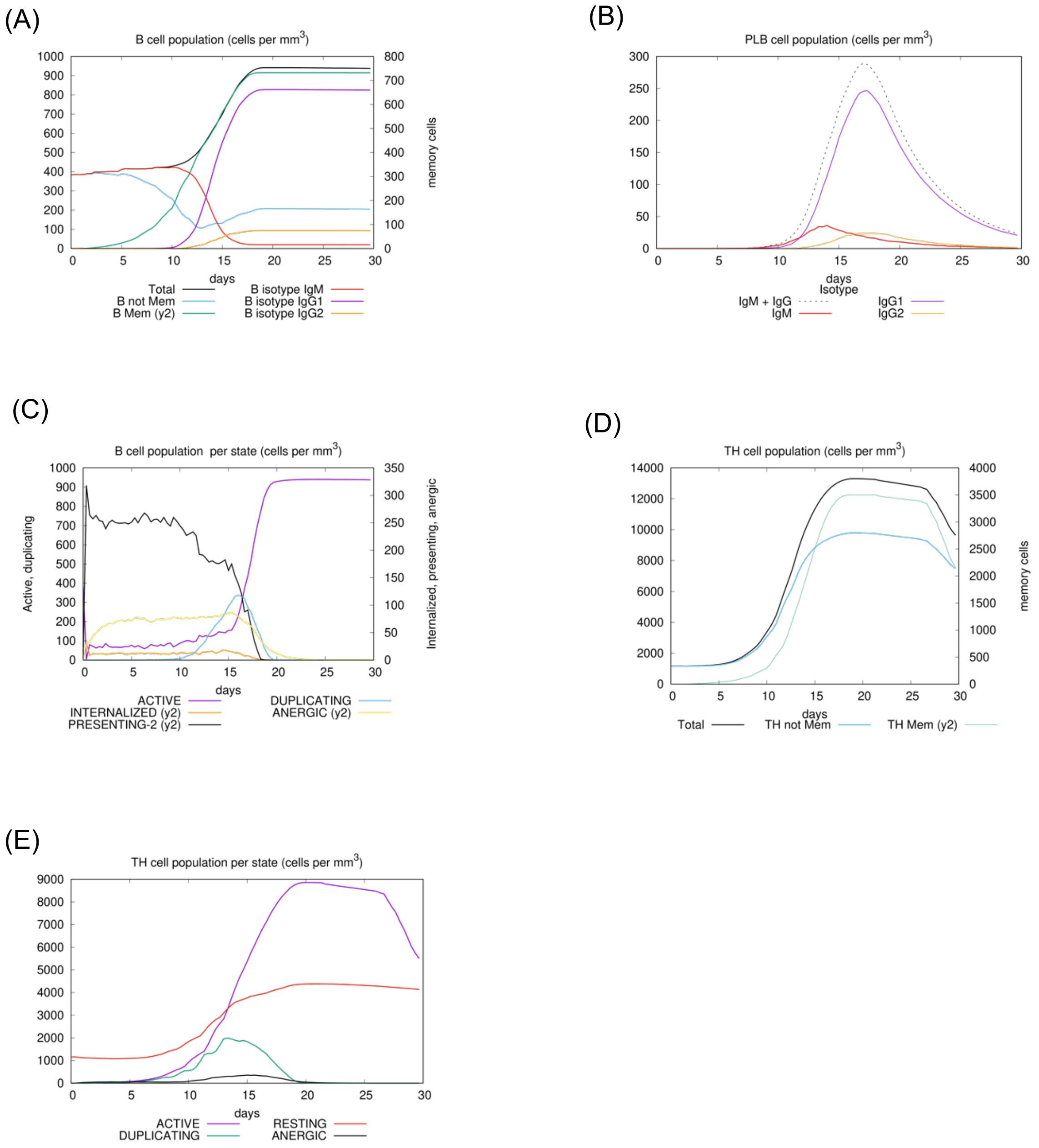

Figure 2 This figure shows details of the population dynamics of B-cells (panel A and C), plasma B cells (PLB in panel B) and helper T lymphocytes (panel D and E).

Panel A of Figure 3 plots the cell counts for CD8 T-cell population (total number, memory cells, not memory). Panel B shows the counts per internal cell state revealing a limited number of anergic cells (numbers on the y2-axis), the early activation of a large number from day 3 (and corresponding decrease of resting cells) entering a duplication phase peaking ad day 5 but progressing through about day 18.

Panels C and D show the total number and the breakout counts for antigen presenting cells DC (dendritic cells in panel C) and MA (that we also indicated as M, macrophages, in panel D). In particular the presentation activity following the internalization of the virus by macrophages terminates at about day 20. Similar behavior is shown in panel E for dendritic cells. Interestingly more macrophages are activated following Rule n.6 (in Appendix A) due to the high level of IFNg released by natural killer cells (cf. Rule n.5 in Appendix A) upon bystander stimulation by danger signals (or damage associated molecules, cf. panel B of Figure 1) secreted by infected cells.

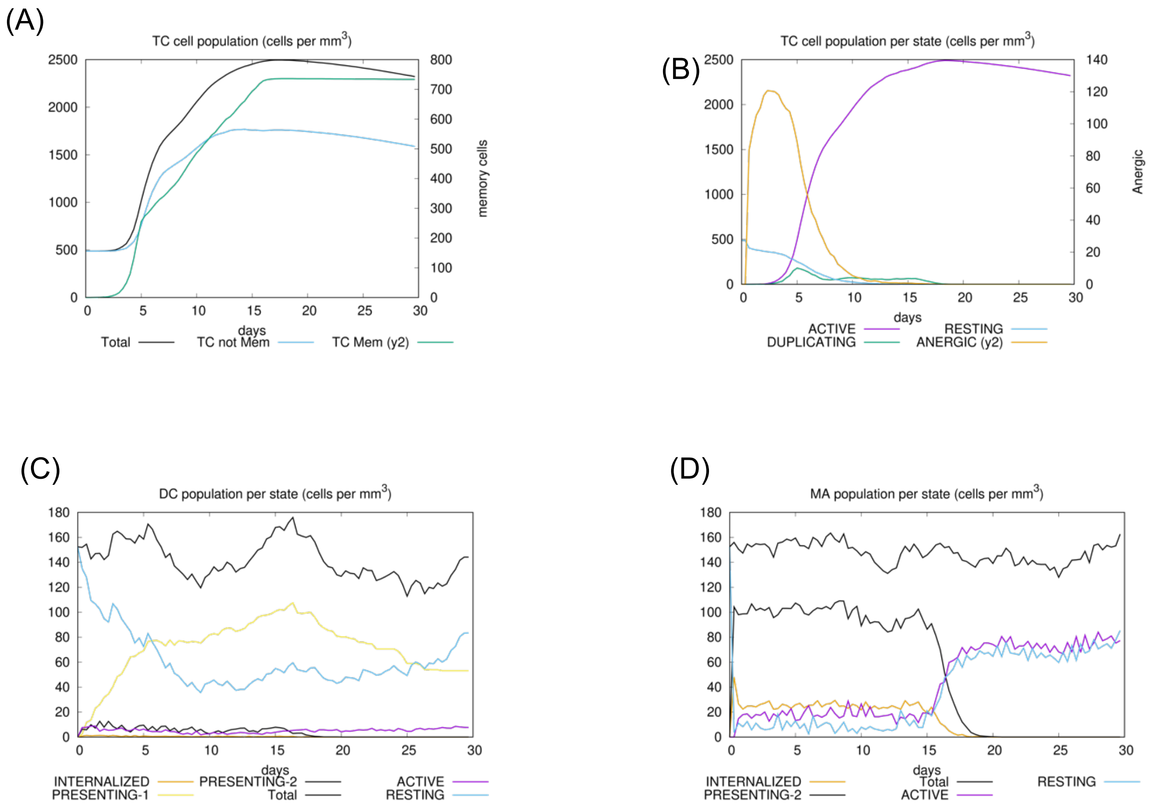

Figure 3 Here we plot the cell counts and detailed intra-cellular state numbers of cytotoxic T cells (panel A and B) and of antigen presenting cells DC and M (respectively in panels C and D). Further details in the text.
